# GEFAAR: a generic framework for the analysis of antimicrobial resistance providing statistics and cluster analyses

**DOI:** 10.1038/s41598-023-44109-3

**Published:** 2023-10-07

**Authors:** Sarah Sandmann, Frieder Schaumburg, Julian Varghese

**Affiliations:** 1 Institute of Medical Informatics, University of Münster, 48149 Münster, Germany; 2 Institute of Medical Microbiology, University of Münster, 48149 Münster, Germany

**Keywords:** Infectious diseases, Health care, Antimicrobial resistance

## Abstract

Easy access to antimicrobial resistance data and meaningful visualization is essential to guide the empirical antimicrobial treatment and to promote the rational use of antimicrobial agents. Currently available solutions are commonly externally hosted, centralized systems. However, there is a need for close monitoring by local analysis tools. To fill this gap, we developed GEFAAR—a generic framework for the analysis of antimicrobial resistance data. Following the example of the German Robert Koch Institute (RKI), an interactive web-application is provided to determine basic pathogen and resistance statistics. In addition to the RKI’s externally maintained database, our application provides a generic framework to import tabular data and to analyze them safely in a local environment. Moreover, our application offers an intuitive web-based user interface to visualize resistance trend analysis as well as advanced cluster analyses on species- or clinic/unit level to generate alerts of potential transmission events.

## Introduction

The emergence of antimicrobial resistance (AMR) is considered a global threat. According to the Centers for Disease Control and Prevention, it was the main cause of death in at least 1.27 million cases worldwide in 2019. Further 5 million deaths were associated with AMR^1^.

To monitor the development of resistance, several surveillance systems are available. The Global Antimicrobial Resistance and Use Surveillance System (GLASS), as an example, was launched by the World Health Organization in 2015^2,3^. It marks the first global collaborative effort aiming at standardized AMR surveillance. Until today, 127 countries are enrolled. GLASS collaborates closely with regional systems like the European Antimicrobial Resistance Surveillance Network (EARS-Net)^4^, the Central Asian and European Surveillance of Antimicrobial Resistance (CAESAR)^5^, the Latin American and Caribbean Network for Antimicrobial Resistance Surveillance (ReLAVRA)^6^, or the Western Pacific Regional Antimicrobial Consumption Surveillance System (WPRACSS)^7^.

The infrastructure is further complemented by additional national surveillance systems, e.g. the Antibiotics Resistance Surveillance (ARS) in Germany. Since 2008, data on detected bacteria and fungi, as well as information on resistance of selected pathogens is provided by the German Robert Koch Institute (RKI). An interactive website allows to generate a report, selecting, among others, the year, species, clinic/unit and specimen of interest^8^.

The RKI provides an externally hosted, centralized database, updated once a year. Currently, data reported by 79 laboratories distributed all over Germany is included. However, an analysis per laboratory is not supported. Instead, only regions like “north-east of Germany” may be chosen. It is not possible to monitor local trends of antimicrobial resistance—within a hospital, or even one or more specific clinics within a hospital. Furthermore, cluster analyses, allowing for detection of regional resistance clusters, cannot be conducted.

To overcome these limitations and to provide an intuitive application for potential usage in every hospital and its subordinated clinical departments around the globe, we developed GEFAAR—a GEneric Framework for the Analysis of Antimicrobial Resistance.

## Methods

### GEFAAR

**Figure 1:**
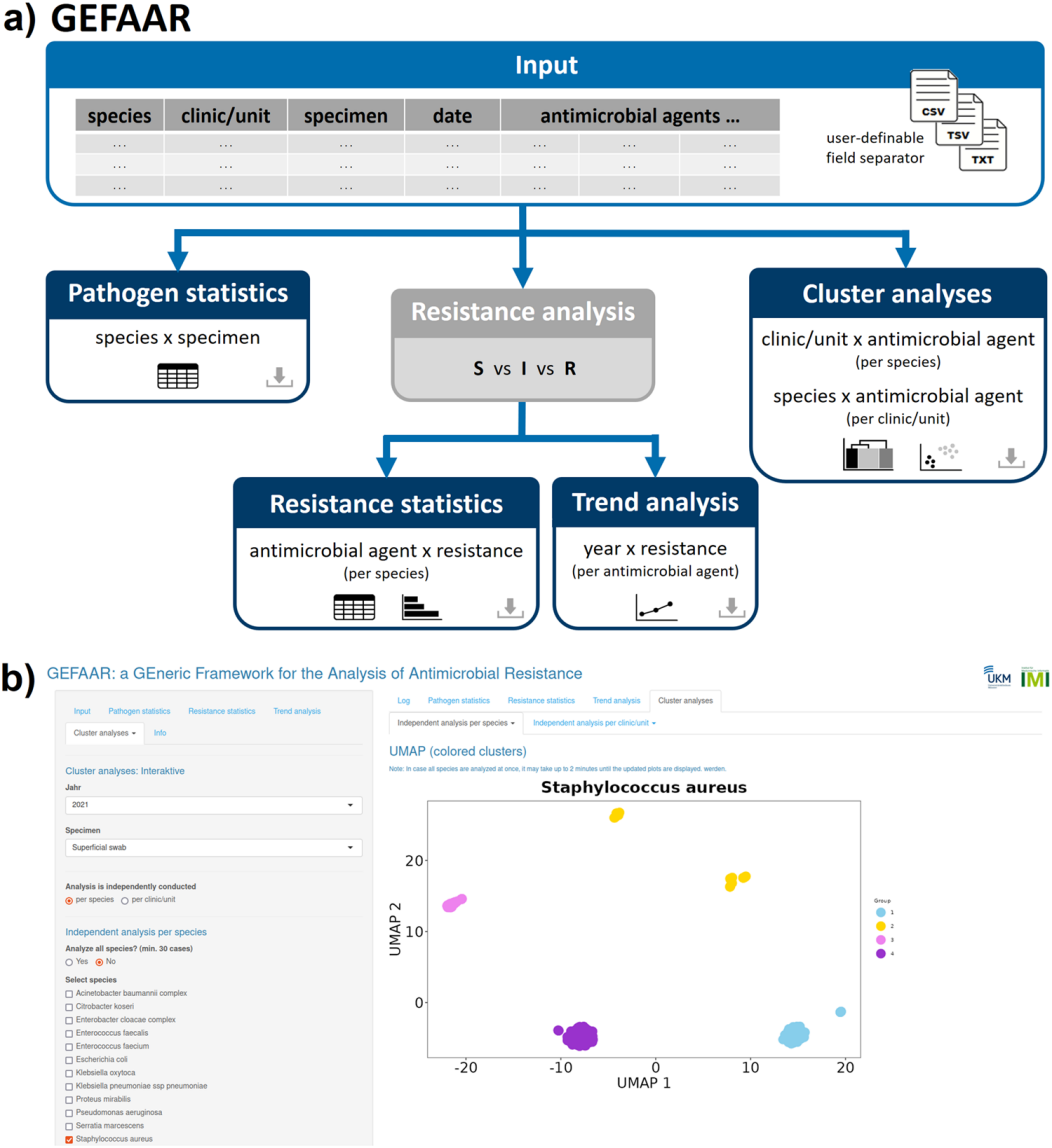
(**a**) Overview of GEFAAR. Based on user-definable input, four analysis modules are available: pathogen statistics, resistance statistics, trend analysis (both based on an automatically conducted resistance analysis) and a set of diverse cluster analyses. *S* susceptible, *I* susceptible with increased exposure, *R* resistant. (**b**) Screenshot of GEFAAR showing the cluster analysis of *Staphylococcus aureus* (year: 2021, specimen: superficial swab) by UMAP as an example.

GEFAAR provides a generic framework to conduct interactive analyses of antimicrobial data, including basic statistics as well as advanced cluster analyses. An overview of the analysis workflow is provided in Fig. 1a, a screenshot in Fig. 1b.

Our software aims at reducing restrictions to input format to a minimum. As a first step, the initial upload is performed. A user selects a file and the corresponding field separator. All common separators (comma, semicolon, tab) are supported. In a subsequent step, a user chooses the columns containing the following metadata: species, clinic/unit, specimen and date. GEFAAR has no restrictions to how the columns are named in the original file (see Supplementary Information, Fig. S1). Different date formats are supported and user-definable. If a user selects a date format that is expected to contradict the data, e.g. selecting ‘dd.mm.yy’, but the input does not contain any ‘.’ in the corresponding column, a note is reported.

GEFAAR assumes that every line in the provided input file corresponds to one isolate. Every column, subsequent to the common metadata columns, provides information on resistance towards one antimicrobial agent. Sticking to common nomenclature, the information is expected to be coded as follows: ‘R’ = resistant, ‘I’ = susceptible with increased exposure, ‘S’ = susceptible, ‘-’ = not analyzed (according to EUCAST^9^).

Our application consists of four main analysis modules: (1) pathogen statistics, (2) resistance statistics, (3) trend analysis, and (4) cluster analyses. The pathogen statistics serve as basic overview of the available data. For a selected year and clinic/unit (optionally: all), an analysis of detected species vs specimen can be conducted. A tabular output is provided.

Both the resistance statistics and the trend analysis are based on a resistance analysis, automatically performed by GEFAAR. In this context, the information on S vs I vs R is processed. For every species and antimicrobial agent, the relative abundance of each category is determined. Performing a detailed analysis of resistance, the 95% confidence intervals are additionally determined, assuming a binomial distribution (*n* being the number of samples per species and antimicrobial agent, *p* being the relative abundance of R; method: Clopper–Pearson intervals^10^). For a selected year, specimen (optionally: all) and clinic/unit (optionally: all), the resistance statistics allow to generate a tabular and graphical overview of antimicrobial agents vs resistance per species. The trend analysis integrates this information on resistance per antimicrobial agent over all available years for a selected species.

In addition, GEFAAR provides the option to execute interactive cluster analyses on one’s input data. A set of diverse clustering approaches is available: ordered heatmaps, hierarchical clustering via heatmap, dimensionality reduction and clustering via Uniform Manifold Approximation and Projection (UMAP)^11^. Hierarchical clustering is one of the most common and well-studied clustering approaches. It is robust, provides detailed information on observations most similar to each other, and is easy to interpret and understand^12^. Dimensionality reduction, on the contrary, provides a more complex approach. Diverse methods are available that allow to transform high-dimensional data to a low-dimensional space. Thus, visualization by means of 2D plots is possible. In GEFAAR, we implemented dimensionality reduction by UMAP. Analyzing high-dimensional single-cell RNA-sequencing data as an example, UMAPs were evaluated as superior to other approaches like principal-component analysis (PCA)^13^ or *t*-distributed stochastic neighbor embedding (t-SNE)^11,14,15^. By the help of UMAPS, detailed molecular characterization of heterogeneous medulloblastoma could be performed, considering four clinically relevant subgroups^16^. Equally, however, UMAPs also enabled to decipher the cellular development of spermatogonia in infertile men^17^. It should however be noted that—as UMAP is a nonlinear dimensionality reduction technique—the axes and exact coordinates in the 2D plots cannot be interpreted as principle components as in PCAs.

Information on resistance vs clinic/unit (per species) as well as resistance vs species (per clinic/unit) can be analyzed for a selected year and specimen (optionally: all). In order to perform successful clustering, data can only contain a limited number of missing values. For ordered heatmaps, we exclude all antimicrobial agents with information on resistance missing in $\ge$ 97% of the samples (analysis per species and per clinic/unit). For hierarchical clustering, we first exclude all antimicrobial agents with information on resistance missing in $\ge$ 70% of the samples. Subsequently, we exclude all samples with information on resistance missing in $\ge$ 70% of the antimicrobial agents (analysis per species and per clinic/unit). As UMAPs can only be generated on even more complete data, stricter filtration has to be applied: First, all antimicrobial agents with information on resistance missing in $\ge$ 20% of the samples are excluded. Subsequently, all samples with any missing information on resistance are filtered. Based on our experience, there is commonly not enough data left for an analysis per clinic/unit due to the strict filtration required by UMAPs. Therefore, UMAP clustering is only implemented for resistance vs clinic/unit (per species).

Clusters are determined using the R package ‘NbClust’^18^. Altogether, NbClust provides 30 different approaches (referred to as indices in NbClust) for determining the optimum number of clusters. However, considering a majority vote over all approaches available would result in a considerably increased run-time. To perform hierarchical clustering, we therefore use the fixed configuration: distance = ‘euclidian’, method = ‘wardD’, index = ‘duda’^19^. To further optimize run-time, a maximum of 5 clusters is considered if < 100 observations are available. Otherwise, a maximum of 10 clusters is considered. In case the algorithm fails to determine an optimum number of clusters, as e.g. no model meets the threshold required by ‘duda’, the message “no clustering possible” is reported.

To determine a stable clustering for UMAPs, we opted for a trade-off between exploring the accordance of assigned clusters using different approaches vs minimum run-time. The following empirically determined clustering strategy is followed: We choose distance = ‘euclidian’ and method = ‘kmeans’. A minimum of 2 clusters, a maximum of 5 is considered. Clustering is performed using the following approaches: silhouette^20^, kl^21^, ch^22^, scott^23^, duda^19^ and dunn^24^. Every approach reports a quality score for each of the possible number of clusters—2, 3, 4 and 5. A reliable clustering is assumed to be available if the following criteria are met: (1) at least two approaches out of kl, ch, scott, duda and dunn report the same number of clusters as optimum. (2) The standard deviation over all quality scores assigned by approach kl to the possible number of clusters—2, 3, 4 and 5—is $\ge$ 5. We assume that a superior clustering is characterized by a peak quality score, clearly differing from the other scores assigned. Thus, a high standard deviation is taken as an indicator for a unique clustering. (3) The standard deviation over all quality scores assigned by approach silhouette is $\ge$ 0.05. The optimum number of clusters is determined based on majority vote. Clusters are assigned according to priority: kl > ch > scott > duda. If this applied approach does not result in a unique clustering result, a corresponding note is displayed.

GEFAAR is programmed in R. A graphical user interface was developed using R Shiny. Interactive elements have been implemented to enable user-friendly operation. All selection menus are continuously updated based on the users’ selection. For example, for a selected specimen, only clinics/units with available data are displayed. Additionally, results of all analyses can be easily exported from within the graphical user interface. The software code, including simulated data, is freely available at https://github.com/sandmanns/gefaar. The R Shiny application can be directly accessed on the public server https://gefaar.uni-muenster.de. The button ‘Load demo data’ allows to simulate and analyze a random set of test data.

### Dataset

In this article, we consider real data from samples collected at the University Hospital Münster (UKM) between 2020 and 2022. The data used in our analysis are routine data, to which we have access based on our daily practice. These data are anonymized. According to the federal law, an informed consent to process these data is not needed (Gesetz zum Schutz personenbezogener Daten im Gesundheitswesen Gesundheitsdatenschutzgesetz—GDSG NW, Paragraph 6). The data set’s main characteristics are summed up in Table 1 (detailed information available in Supplementary Table S1).

For all three years, a comparable number of samples is available. Of note, focusing on an event-based analysis, duplicate isolates were included if the interval between antimicrobial susceptibility testing was $\ge$ 7 days to consider changes of antimicrobial resistances over time^25^. For all years, data based on the same seven specimens are available: blood culture, deep respiratory secretion, deep swab/tissue, foreign body, punctate/secretion, superficial swab and urine. Due to data privacy, all clinics haven been re-named.

## Results

For the interactive analysis of AMR, we developed the generic framework GEFAAR. On September, 1st 2022, it was launched at the UKM. Currently, GEFAAR is used for the analysis of 56,852 samples.

### Pathogen statistics

The pathogen statistics provide count tables for the number of detected species within a selected year, stratified into specimens in which they were detected. Integration over all vs a specific clinic/unit can be chosen. A cut-off value of $\ge$ 30 samples, suggested by GLASS^2^, is enabled by default. Results, showing the top-10 species detected in 2020 vs 2021 vs 2022 over all clinics vs clinic 36 are summed up in Table 2 (screenshots of the interactive output generated with GEFAAR available in Supplementary Information, Figs. S2–S7, exported files containing information on all species are provided as Supplementary Tables S2–S7).

It can be observed that *Escherichia coli* was the most abundant species in samples analyzed at the UKM (2020: 21.4%; 2021: 20.8%; 2022: 18.7%), followed by *Staphylococcus aureus* (10.0% vs 10.3% vs 10.8%) and *Staphylococcus epidermidis* (7.6% vs 7.4% vs 7.2%). For clinic 36, *E. coli* can also be observed as the most abundant species. In second place, however, is *Enterococcus faecium* (rank 6 over all clinics in 2020, rank 7 in 2021 and 2022).

With respect to specimen, considerable species-dependent differences can be observed as one would expect. While *E. coli* is most commonly detected in urine, it is only rarely detected in foreign bodies (e.g. i.v. catheters). However, a slight trend towards increasing proportion in foreign bodies can be observed (2020: 3.3%; 2021: 3.5%; 2022: 5.8%).

### Resistance statistics

For a selected year, specimen (optionally: all), clinic/unit (optionally: all) and species (optionally: all), GEFAAR performs statistical analysis of resistance. A tabular overview of the antimicrobial agents, the frequency of susceptible (S), susceptible with increased exposure (I) and resistant (R) test results^9^, as well as the 95% confidence intervals (CIs) for the resistance rates are generated and provided as ‘data sheet antimicrobial agents’. If data on more than one species is available for the selected configuration, information on all species is reported below each other. In accordance with common practice, evaluation requires $\ge$ 30 isolates per species^2^. In addition, a threshold of 30 is also applied for each antimicrobial agent to ensure validity of the data and a reasonable length of the confidence intervals. To demonstrate the function of GEFAAR, output of the data sheet, providing detailed information on the resistance of *E. coli* towards antimicrobial agents in 2020 vs 2021 vs 2022 (specimen: urine, clinic/unit: all) is provided in Table 3 (screenshots of the interactive output generated with GEFAAR available in Supplementary Information, Figs. S8–S10, files exported from GEFAAR available as Supplementary Tables S8–S10, sheet 2).

By default, data on antimicrobial agents are sorted by decreasing susceptibility. Ertapenem, meropenem and tigecycline all feature the highest susceptibility rates (100%). The high number of available samples leads to especially narrow confidence intervals for carbapenems (i.e. ertapenem and meropenem).

**Figure 2:**
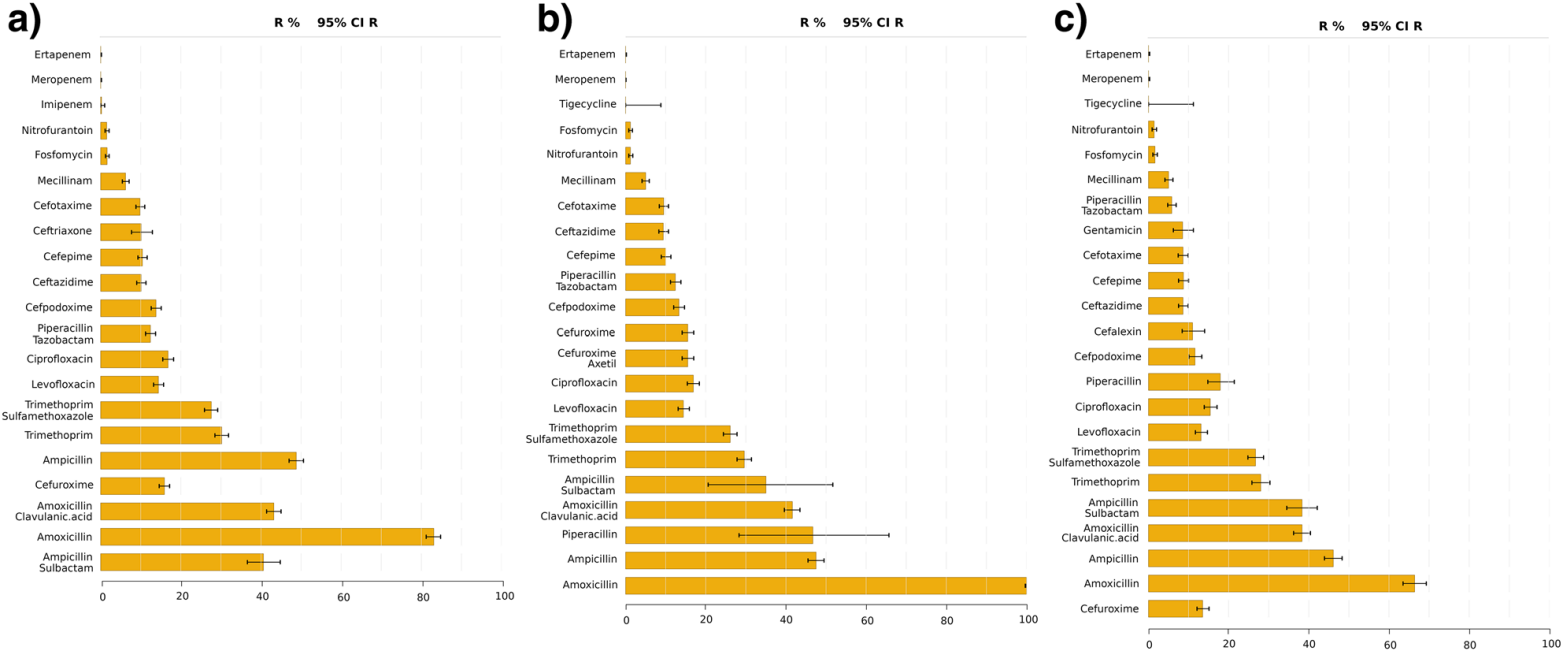
Resistance statistics for *Escherichia coli* (specimen: urine, clinic/unit: all). Barplots visualize the resistance against antimicrobial agents in (**a**) 2020, (**b**) 2021, (**c**) 2022.

In addition to the data sheet, a visual summary of the results is generated, focusing on the resistance rates and their 95% CIs (‘figures antimicrobial agents’). At a glance, the bar plots allow the identification of antimicrobial agents with the lowest proportion of resistant isolates, including confidence of this assessment. By accurately selecting the specimen and clinic/unit, a physician can make a decision based on data that is exactly matching his/her situation. Figures summing up the resistance rates for *E. coli* (specimen: urine, clinic/unit: all) are available in Fig. 2 (screenshots of the interactive output generated with GEFAAR available in Supplementary Information, Figs. S11–S13, files exported from GEFAAR available as Supplementary Tables S8–S10, sheet 3).

### Trend analysis

**Figure 3:**
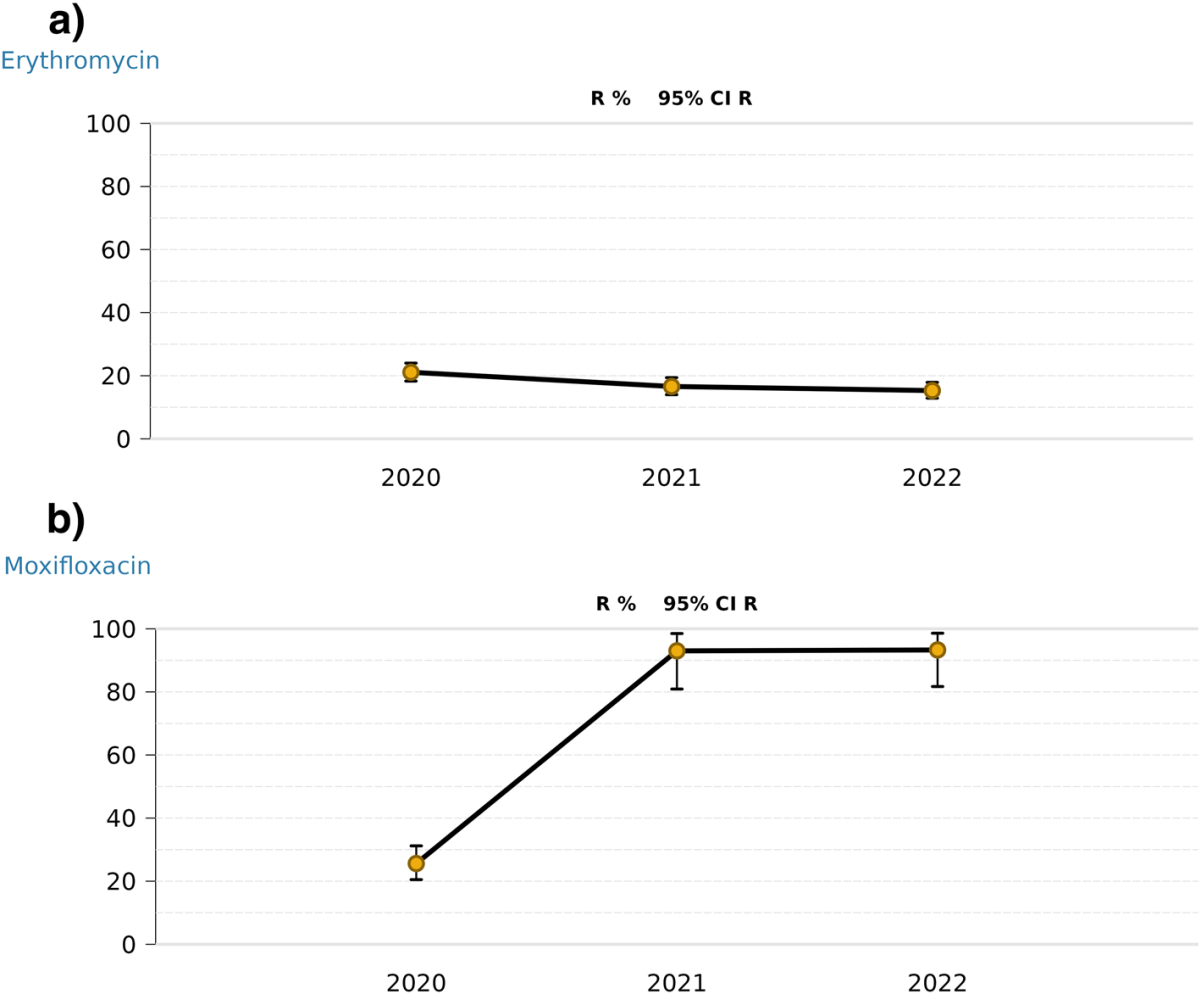
Trend analysis showing the development of resistance of *Staphylococcus aureus* (specimen: superficial swab, clinic/unit: all). (**a**) For erythromycin a minor decrease in resistant isolates can be observed. (**b**) For moxifloxacin data indicates a significant increase in resistance.

While all essential information on resistance is already provided by the resistance statistics, manually changing the selected year and re-analyzing the data to explore the development of resistance over time is tedious. Therefore, we additionally implemented a module for trend analysis to GEFAAR. For a selected specimen (optionally: all), clinic/unit (optionally: all) and species (threshold $\ge$ 30), every antimicrobial agent characterized by sufficient data ($\ge$ 30 samples per year) is analyzed. If one or more years are characterized by insufficient data (< 30 samples), no resistance rate is calculated for the corresponding years. The remaining years, however, are evaluated. The results of a typical trend analysis (specimen: superficial swab, clinic/unit: all, species: *S. aureus*, antimicrobial agents: erythromycin and moxifloxacin) are provided in Fig. 3 (screenshots of the interactive output generated with GEFAAR available in Supplementary Information, Figs. S14–S15, files exported from GEFAAR available as Supplementary Tables S11–S12).

A point diagram with connected lines shows the development of resistance over time. Confidence intervals are added to the plots, just like in case of the resistance statistics. For erythromycin (Fig. 3a), a minor decrease in resistance over time can be observed (2020: R = 21.1%; 2021: R = 16.6%; 2022: R = 15.3%). For moxifloxacin, however, data indicates a considerable increase in resistance (Fig. 3b). In 2020, the estimated resistance rate is R = 25.6% (CI 95% = [20.5–31.2]), while it increased to R = 93.0% (CI 95% = [80.9–98.5]) in the subsequent year. At a glance, visualization by GEFAAR’s trend analysis allows to identify this change in resistance rate as a significant increase.

### Cluster analyses

GEFAAR offers a set of diverse cluster analyses. They allow for detailed evaluation of antimicrobial resistance for a selected year and specimen (optionally: all) to detect and categorize isolates with similar resistance phenotype characteristics. An analysis can be conducted on two levels: (1) per species, and (2) per clinic/unit. All vs a user-definable set of species and clinics/units may be evaluated.

An analysis per species provides the option to explore the relation between clinics/units and antimicrobial agents. Resistance clusters, indicating clonal expansion/outbreaks within one specific or several clinics/units can generally be detected. The following analysis options are available: a heatmap with data ordered by clinic/unit and resistance provides a first overview, identifying clinics/units with increased resistance to one or a combination of several antimicrobial agents. A heatmap with data ordered by clinic/unit and date permits assessment of the development of resistance over time. Thereby, spread of a species with a specific resistance profile may be detected. Common hierarchical clustering and visualization via heatmap is equally supported as more advanced clustering via dimensionality reduction, using UMAPs^11^. While information on clinics/units, antimicrobial agents and resistance are directly available also in clustered heatmaps, it is mainly lacking in UMAPs. For the generated UMAPs, GEFAAR provides the option to color clinics/units (to identify clinic-specific resistance profiles at a glance) as well as clusters. Subsequently, additional heatmaps can be generated, providing information on the UMAP clusters as annotation. Heatmaps can be ordered by cluster or clinic/unit. Thereby, details on the resistance profile per cluster and clinic/unit can be further investigated.

**Figure 4:**
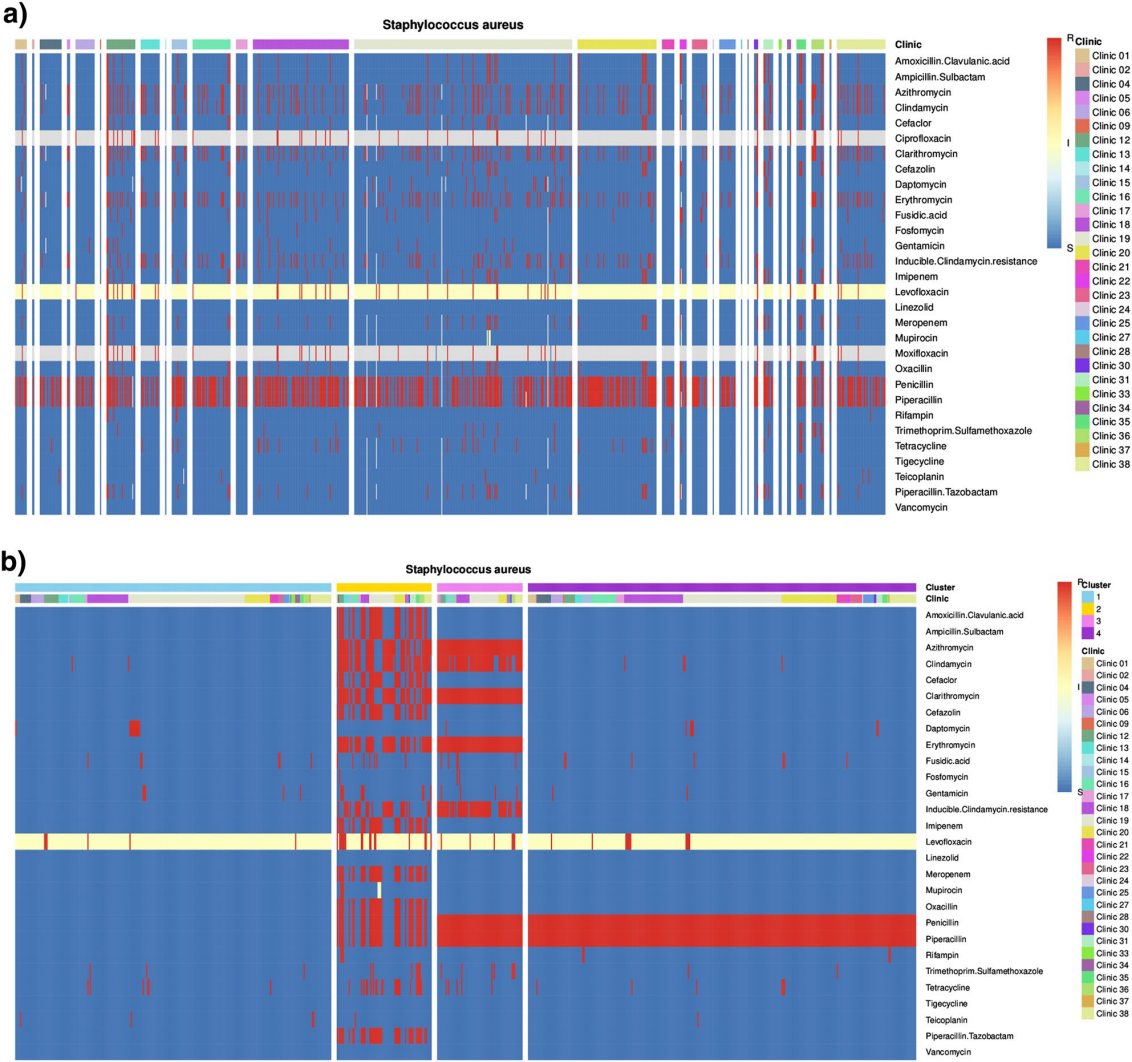
Exported GEFAAR cluster analyses, evaluating data on *Staphylococcus aureus* (year: 2021, specimen: superficial swab). Every column represents one sample, every row one antimicrobial agent. Colors indicate blue: susceptible, yellow: susceptible with induced exposure, red: resistant, grey: no data available. (**a**) Heatmap ordered by 1) clinic/unit and 2) date. (**b**) Heatmap showing data ordered by UMAP clusters and clinic/unit (annotated clusters in top row).

To demonstrate the functionalities of GEFAAR, we performed clustering of *S. aureus* (year: 2021, specimen: superficial swab). Altogether, 747 cases could be evaluated with the selected configuration. A heatmap with data ordered by (1) clinic/unit and (2) date is shown in Fig. 4a, a heatmap with annotated UMAP clusters, ordered by cluster and clinic/unit is shown in Fig. 4b (heatmap with data ordered by (1) clinic/unit and (2) resistance available in Supplementary Information, Fig. S16; UMAP with colored clinics/units in Fig. S17; UMAP with colored clusters in Fig. S18; heatmap with data ordered by clinics/units and annotated UMAP clusters in Fig. S19; hierarchical clustering could not be conducted; cluster analyses exported from GEFAAR available as Supplementary Data S1).

For the heatmap ordered by clinic/unit and date (Fig. 4a), data on 30 clinics and 30 antimicrobial agents is available. As only lenient filtering for missing data is applied, some antimicrobials are included despite featuring a relatively high level of missing data (95% missing for ciprofloxacin, 94% for moxifloxacin). It can be observed that samples characterized by resistance towards one or more antimicrobial agents are randomly distributed across the different clinics. An accumulation of resistance over the year cannot be observed.

Clustering by dimensionality reduction (UMAP) requires strict filtration of missing values. As a consequence, ciprofloxacin and moxifloxacin had to be excluded from further analysis of *S. aureus* clusters. Analysis by UMAP shows a clear separation of data (Supplementary Information, Fig. S17). Clustering suggests presence of four distinct clusters, each of them characterized by a specific resistance profile (see Fig. 4b): Cluster 1 is classical penicillin-susceptible *S. aureus*. Susceptibility to all relevant antibiotics can be observed. Cluster 4 is typical penicillin-resistant, but oxacillin-susceptible *S. aureus*, reflecting the marked increase in penicillin-resistance in the past century. Isolates in cluster 3 show resistance to penicillin and also to azithromycin, clarithromycin, erythromycin and piperacillin. In most cases, resistance to clindamycin can additionally be observed. While clusters 1, 3, and 4 reflect fairly typical *S. aureus* that can also observed in the community, cluster 2 unites diverse isolates with considerably more resistances. Two subclusters can be observed in both the UMAP and the heatmap that can be distinguished as oxacillin-resistant (MRSA) vs oxacillin-susceptible (MSSA). Considering clinics (annotation in second row), no association with any of the four clusters can be observed. Thus, our results indicate that no outbreak—especially of multiresistent *S. aureus*—has taken place.

**Figure 5:**
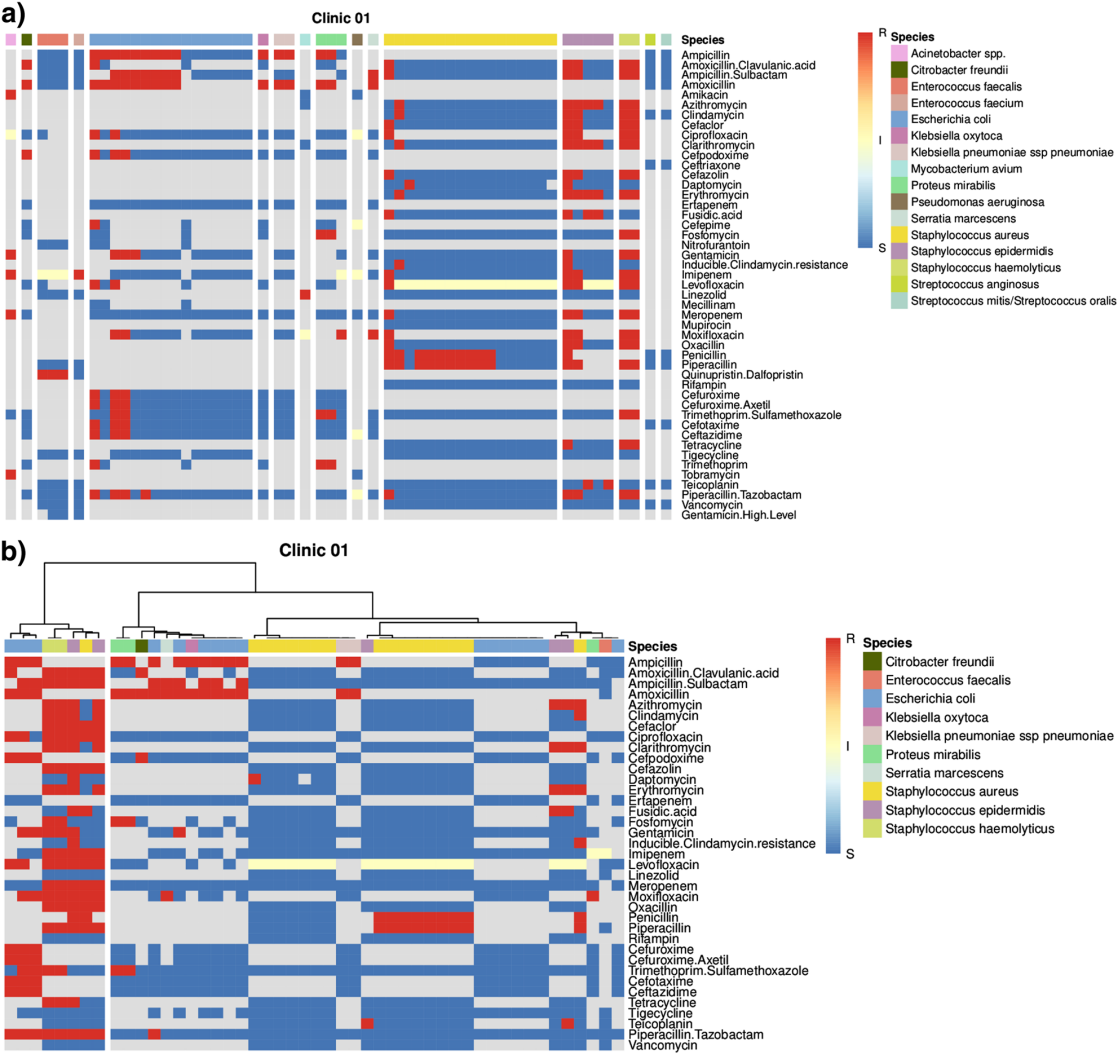
Exported GEFAAR cluster analyses, evaluating data on clinic 01 (year: 2021, specimen: all). Every column represents one sample, every row one antimicrobial agent. Colors indicate blue: susceptible, yellow: susceptible with induced exposure, red: resistant, grey: no data available. (**a**) Heatmap ordered by species. (**b**) Heatmap with hierarchical clustering.

To perform an in-depth analysis of the relation between species vs resistance, clustering on clinic-/unit-level is supported. We performed analysis of clinic 01 (year: 2021, specimen: all). A heatmap with data ordered by species is available in Fig. 5a, hierarchical clustering in Fig. 5b (cluster analyses exported from GEFAAR available as Supplementary Data S2).

For the heatmap ordered by species (Fig. 5a), data on 16 species and 47 antimicrobial agents is displayed. Due to lenient filtration for missing data, species like *Mycobacterium avium*, characterized by 89% missing data, are included in this general overview. With respect to hierarchical clustering (Fig. 5b), we exclude species and samples with $\ge$ 70% missing data. As a result, information on only 10 species and 37 antimicrobials remains. Analysis reveals two distinct clusters, characterized by different resistance profiles. However, no major patterns, crossing the species boundaries, can be observed.

## Discussion

In this work, we introduced GEFAAR—a novel, generic approach for assessing AMR in individual hospitals. To the best of our knowledge, GEFAAR is the first application providing not just common pathogen and resistance statistics, but also an easy-to-use interface to perform trend analysis as well as advanced cluster analyses.

It may be argued that a plethora of systems to monitor and analyze AMR already exist. In their systematic review in 2020, Diallo et al.^26^ identified 71 surveillance systems. However, these systems are commonly maintained externally. The information they analyze and display differs, partly considerably. Furthermore, systems are mainly available in developed countries.

Recently, the R package ‘AMR’ was published to ease working with data on antimicrobial resistance^27^. An extensive set of functions is available, e.g. filtering data, calculating antimicrobial resistance or determining a regression model to predict future AMR. However, the software is—primarily—a statistical software. Despite providing several tutorials, advanced programming skills are inevitably required to perform analyses with the R package AMR, including the export of tables or plots exceeding the implemented bar plot option.

We hold the view that a surveillance system is only best if it is tailored to local needs and easy to use to increase acceptance. For this reason, GEFAAR was developed in close collaboration with end-users. Following their requests and suggestions, we implemented an intuitive, user-friendly interface. For the pathogen and resistance statistics, we took our cue from the well-established ARS of the RKI—Germany’s public health institute. We developed the configuration panel, the interactive results as well as the Excel export following the RKI example. However, we added further features to this basic interface based on user feedback, e.g. reporting the pathogen statistics for all specimens separately, in addition to total counts. In the same design, we implemented a trend analysis and a set of cluster analyses. Heatmaps allow for visualizing a large amount of information in a clear way. Hierarchical clustering was chosen as a relatively easy and comprehensive way of clustering. Dimensionality reduction and clustering via UMAP was selected as a more advanced clustering approach, providing an option to explore complex patterns of resistance in the high-dimensional data we are dealing with.

While surveillance systems like the German ARS provide database updates only once a year, in GEFAAR, we implemented an upload option. Minimum input format requirements allow to analyze a hospital’s routine data with respect to AMR. Thereby, GEFAAR provides an easy-to-use option to study AMR including small hospitals in rural areas and developing countries that are often not considered by the common national and international ARS. Furthermore, as GEFAAR allows for the immediate analysis of data, it provides the framework for early detection of emerging AMR clusters so that quick action can be taken.

Programming knowledge is not required for any of the analyses to be conducted with GEFAAR. At the UKM, a local server was set up to run our software. Thereby, it can be reached within the hospital’s intranet with any web-browser as a simple interactive website. No tools have to be installed. Additionally, all data uploaded to GEFAAR for analysis are securely kept within the hospital. As an alternative, GEFAAR can also be run on a local computer, requiring only an installation of R. The software code is freely available at https://github.com/sandmanns/gefaar. In addition, the web-application can be directly accessed on the public server https://gefaar.uni-muenster.de. The infection prevention and control (IPC) board of the UKM has advised all prescribers the use of GEFAAR.

As future work, we plan to extend functionalities of GEFAAR. Regarding resistance statistics and trend analysis, options for additionally including data from public databases like the ARS of the RKI will be examined. This would allow a user to better classify the results. Possible bias caused by selection of the samples and tests, leading to an overestimation of resistance compared to the average population, could be investigated. With regard to cluster analyses, we will explore further analyses that, for example, look more closely at the association of clinics/units with resistance clusters. Additionally, alternative clustering approaches and configurations will be further explored, including our algorithm estimating the optimum number of clusters, the minimum and the maximum number of clusters considered.

Concluding, GEFAAR represents a novel option for the interactive analysis of AMR, providing basic statistic as well as advanced cluster analyses. Due to its generic framework, tabular data can be imported and analyses conducted independent of externally maintained databases. Thereby, GEFAAR provides guidance for empirical antimicrobial therapy and support to detect AMR clusters within or beyond clinics/units if other platforms are not available (e.g. whole genome sequencing).

**Table 1:** Overview of the real dataset analyzed with GEFAAR.

	2020	2021	2022	Unique	Overlap
					$$2020\cap 2021\cap 2022$$
Samples	20,743	18,604	17,505	–	–
Patients	7827	7488	6860	–	–
Specimens	7	7	7	7	7
Clinics	36	35	33	38	32
Species	306	238	229	391	153
Antimicrobial agents	81	87	81	97	66

Samples were collected at the University Hospital Münster between 2020 and 2022.

**Table 2:** Pathogen statistics of the top-10 species stratified into specimens in which they were detected (years: 2020 vs 2021 vs 2022, clinic/unit: all vs clinic 36, cut-off 30 cases: enabled).

Year	Clinic/unit	Species	Blood culture N%	Deep respiratory secretion N%	Deep swab/tissue N%	Foreign body N%	Punctate/secretion N%	Superficial swab N%	Urine N%	Sum N%
2020	All	*E. coli*	12.5	12.6	9.3	3.3	12.5	11.4	43.7	21.4
		*S. aureus*	6.5	18.2	11	6.6	4	26.6	1.4	10
		*S. epidermidis*	28	0	12.4	39.8	6.5	3	0.3	7.6
		*P. aeruginosa*	2.3	8.7	5.8	3.5	4.3	11.9	5.9	6.6
		*E. faecalis*	2.9	0	7	5.8	8.3	2.1	8	5.5
		*E. faecium*	6.9	0	8.3	7.3	11.3	1.7	4.8	5.3
		*K. pneumoniae*	3.3	5.2	2.1	1.5	4	3.5	8.7	5
		*E. cloacae*	1.7	7.6	3.5	2.5	4.4	4.6	3.4	4
		*P. mirabilis*	1	3	1.8	0.9	1	4.5	6.5	3.7
		*K. oxytoca*	1.1	5.2	1	0.5	1.7	2.3	3.6	2.5
2021	All	*E. coli*	13.3	12.4	10.2	3.5	10.7	10	42.6	20.8
		*S. aureus*	7.8	18.9	10.5	8.2	3	25.1	1.9	10.3
		*S. epidermidis*	23.9	0	13	36.1	7.3	5.5	0.4	7.4
		*P. aeruginosa*	2.6	8.6	4	2.9	4	10	5.6	5.9
		*E. faecalis*	3.4	0	7	6.4	7.6	2.7	8.3	5.6
		*K. pneumoniae*	2.7	7.6	2.7	2.3	3	3.8	8.2	5.2
		*E. faecium*	7.7	0.1	6.6	6.4	11.7	2	5.4	5.1
		*P. mirabilis*	1.2	3.3	1.8	1	0.6	4.2	6.5	3.7
2022	All	*E. coli*	15.8	11.3	8.2	5.8	10.2	7.6	42.7	18.7
		*S. aureus*	6.9	17.6	9.8	6.9	4.2	26.3	1.8	10.8
		*S. epidermidis*	20.3	0	12.4	31.9	7.4	6.4	0	7.2
		*P. aeruginosa*	2.1	10	3.6	4.8	1.3	10.2	5.9	5.9
		*K. pneumoniae*	4.6	6.7	3	2.4	3.8	3.7	9.8	5.6
		*E. faecalis*	3	0	6.9	5.2	9.9	2.2	6.7	5.1
		*E. faecium*	6.8	0	5.5	3.8	10.8	1.1	5.1	4.4
		*E. cloacae*	1.7	6.6	3.4	2.4	4	5.1	3.6	4
2020	Clinic 36	*E. coli*	16.6	9.7	7.9	5	14.3	22.5	44.3	25
		*S. epidermidis*	33.2	0	14.3	46.7	17.1	2.3	0	13.4
		*E. faecium*	9.2	0	7.9	3.3	8.6	2.3	19	10.4
		*S. aureus*	3	21.5	6.3	8.3	5.7	11.6	1.2	6.1
		*E. faecalis*	1.2	0	6.3	3.3	5.7	3.1	11.1	5.2
		*P. aeruginosa*	1.5	6.2	12.7	1.7	0	16.3	4.3	5.2
		*K. pneumoniae*	1.8	6.2	3.2	1.7	5.7	2.3	5.3	3.8
		*S. haemolyticus*	6.5	0	3.2	11.7	0	0	0	2.6
2021	Clinic 36	*E. coli*	15.4	5.4	11.5	2.3	22	13.7	38.3	21.2
		*E. faecium*	11.8	0	11.5	6.8	2.4	2.9	21.2	11.6
		*S. epidermidis*	29.9	0	3.8	50	17.1	5.9	0.3	10.4
		*S. aureus*	0.4	22.1	9.6	0	2.4	12.7	0.8	6.2
		*P. aeruginosa*	2.8	15.2	7.7	6.8	4.9	7.8	3	6.1
		*K. pneumoniae*	2	4.9	3.8	6.8	0	6.9	9.6	5.9
		*E. faecalis*	1.2	0	5.8	6.8	2.4	4.9	9.6	4.8
		*S. haemolyticus*	8.7	0	3.8	13.6	2.4	1	1.3	3.4
2022	Clinic 36	*E. coli*	15.2	5.7	9.4	6.7	10.2	9.5	42.2	21.3
		*E. faecium*	13.9	0	6.2	6.7	14.3	0	14.5	9.3
		*S. epidermidis*	24.2	0	12.5	66.7	8.2	7.6	0.6	8.5
		*S. aureus*	2.6	21.3	6.2	13.3	2	20	2	8.3
		*K. pneumoniae*	2.6	3.8	6.2	0	2	11.4	10.1	6.5
		*P. aeruginosa*	3.9	11.4	6.2	0	0	10.5	3.9	6
		*E. faecalis*	1.3	0	10.9	0	12.2	2.9	6.1	4
		*P. mirabilis*	0	3.3	3.1	0	0	3.8	5	3

*E. coli*: *Escherichia coli*; *S. aureus*: *Staphylococcus aureus*; *S. epidermidis*: *Staphylococcus epidermidis*; *P. aeruginosa*: *Pseudomonas aeruginosa*; *E. faecalis*: *Enterococcus faecalis*; *E. faecium*: *Enterococcus faecium*; *K. pneumoniae*: *Klebsiella pneumoniae ssp pneumoniae*; *E. cloacae*: *Enterobacter cloacae complex*; *P. mirabilis*: *Proteus mirabilis*; *K. oxytoca*: *Klebsiella oxytoca*; *S. haemolyticus*: *Staphylococcus haemolyticus*.

**Table 3:** Resistance statistics: data sheet antimicrobial agents for *Escherichia coli* comparing 2020 vs 2021 vs 2022 (specimen: urine, clinic/unit: all).

Year	Antimicrobial agents	N	S %	I %	R %	95% CI R
2020	Ertapenem	2928	100.0	0.0	0.0	0.0–0.2
	Meropenem	2933	100.0	0.0	0.0	0.0–0.2
	Imipenem	573	99.7	0.2	0.2	0.0–1.0
	Nitrofurantoin	2911	98.5	0.0	1.5	1.1–2.1
	Fosfomycin	2914	98.4	0.0	1.6	1.2–2.1
	Mecillinam	2914	93.8	0.0	6.2	5.4–7.1
	Cefotaxime	2927	90.2	0.0	9.8	8.8–11.0
	Ceftriaxone	556	89.9	0.0	10.1	7.7–12.9
	Cefepime	2884	89.6	0.0	10.4	9.3–11.6
	Ceftazidime	2925	89.1	0.8	10.1	9.0–11.3
	Cefpodoxime	2924	86.2	0.0	13.8	12.6–15.1
	Piperacillin.Tazobactam	2888	85.5	2.1	12.4	11.2–13.7
	Ciprofloxacin	2935	79.6	3.6	16.8	15.5–18.2
	Levofloxacin	2922	74.7	10.9	14.4	13.2–15.7
	Trimethoprim.Sulfamethoxazole	2925	72.4	0.1	27.6	25.9–29.2
	Trimethoprim	2911	69.8	0.0	30.2	28.5–31.9
	Ampicillin	2934	47.1	4.1	48.8	47.0–50.6
	Cefuroxime	2926	40.0	44.2	15.9	14.6–17.2
	Amoxicillin.Clavulanic.acid	2916	28.2	28.6	43.2	41.4–45.0
	Amoxicillin	1677	10.0	6.9	83.1	81.2–84.8
	Ampicillin.Sulbactam	571	4.9	54.5	40.6	36.6–44.8
2021	Ertapenem	2502	100	0	0	0.0–0.2
	Meropenem	2524	100	0	0	0.0–0.1
	Tigecycline	40	100	0	0	0.0–8.8
	Fosfomycin	2496	98.8	0	1.2	0.8–1.7
	Nitrofurantoin	2495	98.8	0	1.2	0.8–1.8
	Mecillinam	2496	95	0	5	4.1–5.9
	Cefotaxime	2507	90.5	0	9.5	8.4–10.7
	Ceftazidime	2500	90.2	0.4	9.4	8.3–10.7
	Cefepime	2473	89.9	0.1	10	8.9–11.3
	Piperacillin.Tazobactam	2500	87.2	0.3	12.4	11.2–13.8
	Cefpodoxime	2505	86.7	0	13.3	12.0–14.7
	Cefuroxime	2505	84.5	0	15.5	14.1–17.0
	Cefuroxime.Axetil	2505	84.5	0	15.5	14.1–17.0
	Ciprofloxacin	2521	80.5	2.6	16.9	15.4–18.4
	Levofloxacin	2492	75.1	10.5	14.4	13.1–15.9
	Trimethoprim.Sulfamethoxazole	2523	73.9	0	26.1	24.4–27.8
	Trimethoprim	2496	70.4	0	29.6	27.8—31.4
	Ampicillin.Sulbactam	40	65	0	35	20.6–51.7
	Amoxicillin.Clavulanic.acid	2494	58.4	0	41.6	39.6–43.5
	Piperacillin	30	53.3	0	46.7	28.3–65.7
	Ampicillin	2521	52.5	0	47.5	45.5–49.5
	Amoxicillin	1172	0	0	100	99.7–100.0
2022	Ertapenem	2068	100	0	0	0.0–0.3
	Meropenem	2080	100	0	0	0.0–0.3
	Tigecycline	31	100	0	0	0.0–11.2
	Nitrofurantoin	2059	98.6	0	1.4	0.9–2.0
	Fosfomycin	2060	98.4	0	1.6	1.1–2.2
	Mecillinam	2060	95	0	5	4.1–6.1
	Piperacillin.Tazobactam	2063	94.1	0.1	5.8	4.8–6.9
	Gentamicin	531	91.5	0	8.5	6.2–11.2
	Cefotaxime	2067	91.4	0	8.6	7.4–9.9
	Cefepime	2045	91.2	0.1	8.7	7.5–10.0
	Ceftazidime	2061	90.9	0.4	8.6	7.5–9.9
	Cefalexin	519	89	0	11	8.4–14.0
	Cefpodoxime	1548	88.4	0	11.6	10.1–13.3
	Piperacillin	542	82.1	0	17.9	14.8–21.4
	Ciprofloxacin	2080	81.1	3.5	15.4	13.9–17.1
	Levofloxacin	2063	76.7	10.1	13.1	11.7–14.7
	Trimethoprim.sulfamethoxazole	2078	73.2	0.1	26.7	24.8–28.7
	Trimethoprim	1540	72	0	28	25.8–30.3
	Ampicillin.Sulbactam	648	61.7	0	38.3	34.5–42.1
	Amoxicillin.Clavulanic.acid	2056	56.9	4.9	38.3	36.2–40.4
	Ampicillin	2080	53.9	0	46.1	43.9–48.3
	Amoxicillin	1048	33.6	0	66.4	63.5–69.3
	Cefuroxime	2067	8.2	78.3	13.5	12.1–15.1

*N* number of samples, *S%* rate of susceptibility, *I%* rate of susceptibility increased exposure, *R%* rate of resistance, *95% CI R* 95% confidence intervals for rate of resistance.

### Supplementary Information

Supplementary Information.

## Data Availability

GEFAAR, including simulated data, is freely available at https://github.com/sandmanns/gefaar. Results of all analyses conducted with GEFAAR during this study are included in this published article and its Supplementary Information files. The dataset analysed during the current study is available from the corresponding author on reasonable request.
