# Supplementary material for: GEFAAR: a generic framework for the analysis of antimicrobial resistance providing statistics and cluster analyses
Source: Sci Rep. 2023 Oct 7;13:16922. doi: 10.1038/s41598-023-44109-3 (PMC10560266; doi:10.1038/s41598-023-44109-3)
Supplement: Supplementary file 1 — Supplementary Information. [file 41598_2023_44109_MOESM1_ESM.zip › SupplementaryFiles/SupplementaryFiles/Supplementary Information.pdf]

Supplementary Information

GEFAAR: a generic framework for the analysis of antimicrobial resistance providing statistics and cluster analyses

Contents

1 Additional methods **3**
1.1 Input 3

2 Additional results **4**
2.1 Pathogen statistics 4
2.2 Resistance statistics 7
2.3 Trend analysis 10
2.4 Cluster analyses 11

3 Legend to supplementary tables **13**

4 Legend to supplementary data **14**

1 Additional methods

1.1 Input

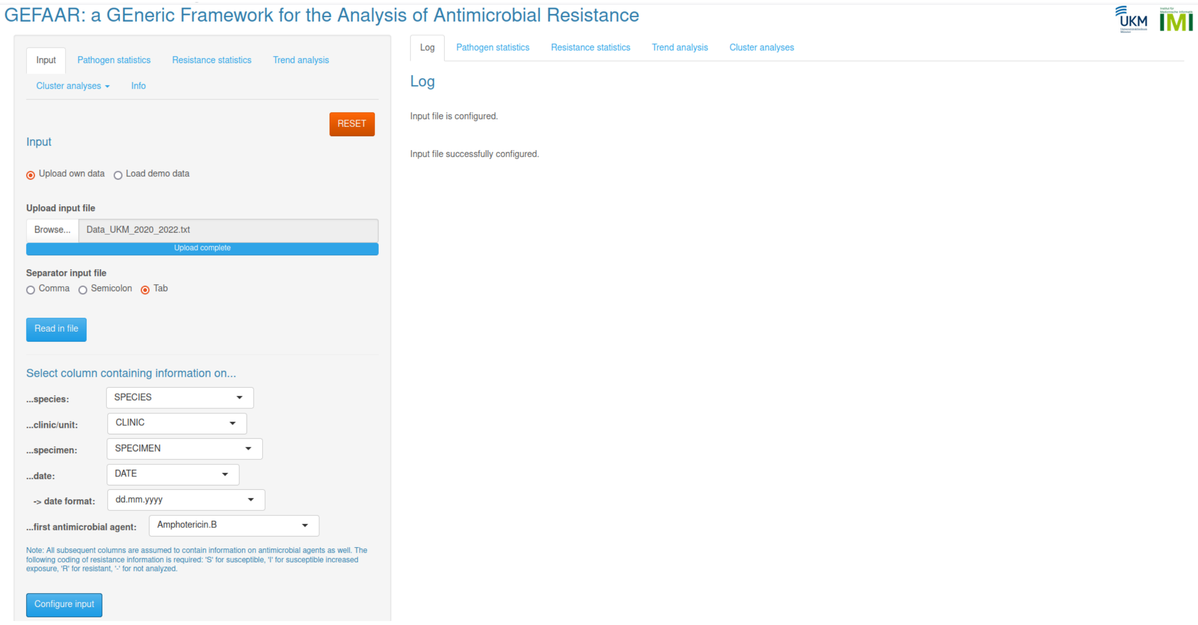

Figure S1: Screenshot from GEFAAR, showing the input of a typical file.

2 Additional results

2.1 Pathogen statistics

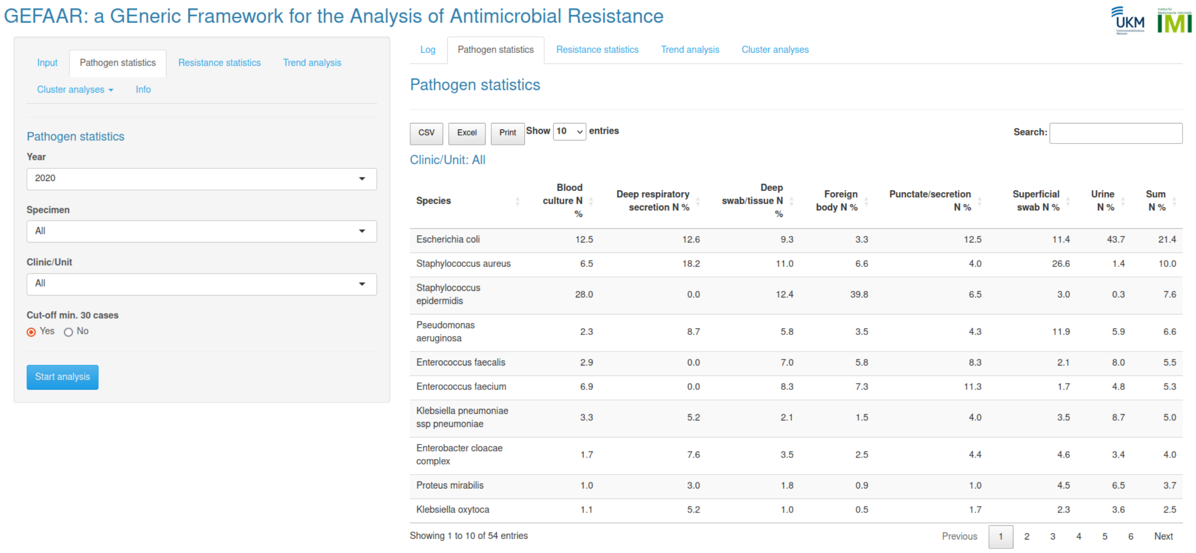

Species	Blood culture N %	Deep respiratory secretion N %	Deep swab/tissue N %	Foreign body N %	Punctate/secretion N %	Superficial swab N %	Urine N %	Sum N %
Escherichia coli	12.5	12.6	9.3	3.3	12.5	11.4	43.7	21.4
Staphylococcus aureus	6.5	18.2	11.0	6.6	4.0	26.6	1.4	10.0
Staphylococcus epidermidis	28.0	0.0	12.4	39.8	6.5	3.0	0.3	7.6
Pseudomonas aeruginosa	2.3	8.7	5.8	3.5	4.3	11.9	5.9	6.6
Enterococcus faecalis	2.9	0.0	7.0	5.8	8.3	2.1	8.0	5.5
Enterococcus faecium	6.9	0.0	8.3	7.3	11.3	1.7	4.8	5.3
Klebsiella pneumoniae ssp pneumoniae	3.3	5.2	2.1	1.5	4.0	3.5	8.7	5.0
Enterobacter cloacae complex	1.7	7.6	3.5	2.5	4.4	4.6	3.4	4.0
Proteus mirabilis	1.0	3.0	1.8	0.9	1.0	4.5	6.5	3.7
Klebsiella oxytoca	1.1	5.2	1.0	0.5	1.7	2.3	3.6	2.5

Figure S2: Screenshot from GEFAAR, showing the pathogen statistics for the top-10 species stratified into specimens in which they were detected (year: 2020, clinic/unit: all).

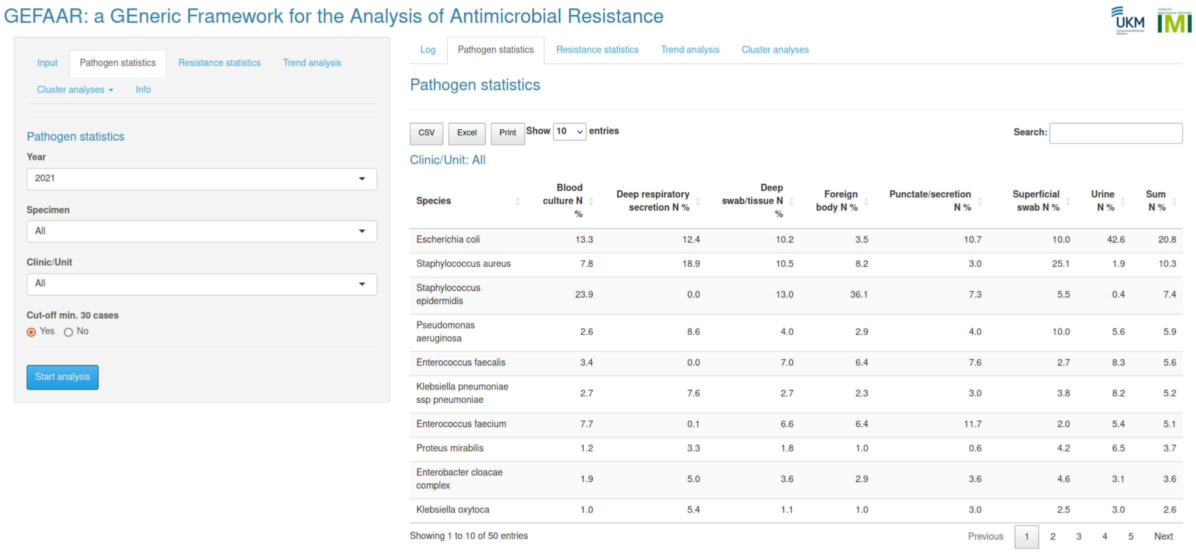

Species	Blood culture N %	Deep respiratory secretion N %	Deep swab/tissue N %	Foreign body N %	Punctate/secretion N %	Superficial swab N %	Urine N %	Sum N %
Escherichia coli	13.3	12.4	10.2	3.5	10.7	10.0	42.6	20.8
Staphylococcus aureus	7.8	18.9	10.5	8.2	3.0	25.1	1.9	10.3
Staphylococcus epidermidis	23.9	0.0	13.0	36.1	7.3	5.5	0.4	7.4
Pseudomonas aeruginosa	2.6	8.6	4.0	2.9	4.0	10.0	5.6	5.9
Enterococcus faecalis	3.4	0.0	7.0	6.4	7.6	2.7	8.3	5.6
Klebsiella pneumoniae ssp pneumoniae	2.7	7.6	2.7	2.3	3.0	3.8	8.2	5.2
Enterococcus faecium	7.7	0.1	6.6	6.4	11.7	2.0	5.4	5.1
Proteus mirabilis	1.2	3.3	1.8	1.0	0.6	4.2	6.5	3.7
Enterobacter cloacae complex	1.9	5.0	3.6	2.9	3.6	4.6	3.1	3.6
Klebsiella oxytoca	1.0	5.4	1.1	1.0	3.0	2.5	3.0	2.6

Figure S3: Screenshot from GEFAAR, showing the pathogen statistics for the top-10 species stratified into specimens in which they were detected (year: 2021, clinic/unit: all).

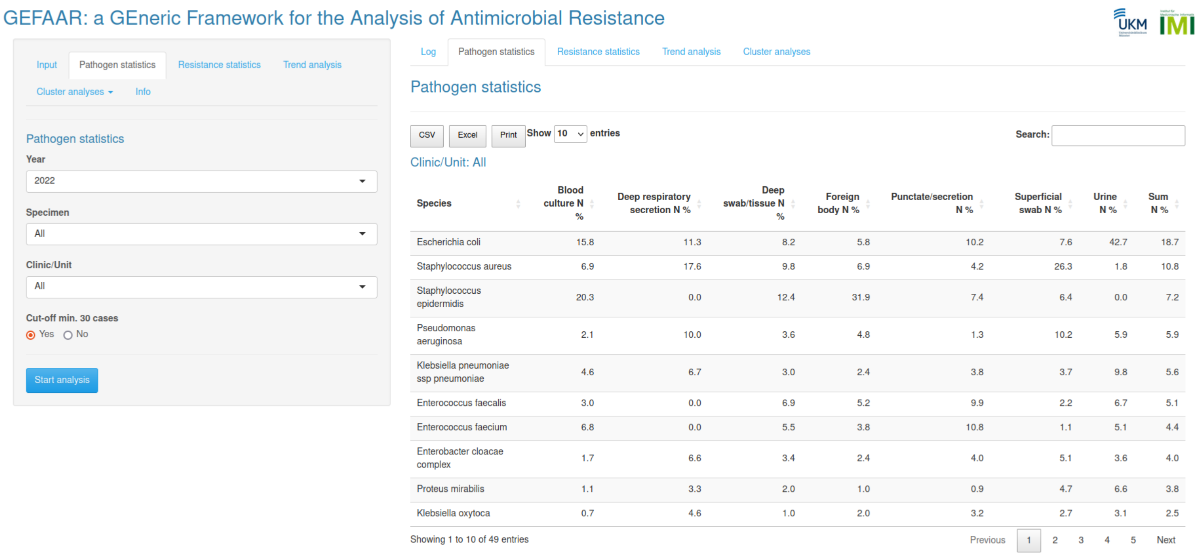

Species	Blood culture N %	Deep respiratory secretion N %	Deep swab/tissue N %	Foreign body N %	Punctate/secretion N %	Superficial swab N %	Urine N %	Sum N %
Escherichia coli	15.8	11.3	8.2	5.8	10.2	7.6	42.7	18.7
Staphylococcus aureus	6.9	17.6	9.8	6.9	4.2	26.3	1.8	10.8
Staphylococcus epidermidis	20.3	0.0	12.4	31.9	7.4	6.4	0.0	7.2
Pseudomonas aeruginosa	2.1	10.0	3.6	4.8	1.3	10.2	5.9	5.9
Klebsiella pneumoniae ssp pneumoniae	4.6	6.7	3.0	2.4	3.8	3.7	9.8	5.6
Enterococcus faecalis	3.0	0.0	6.9	5.2	9.9	2.2	6.7	5.1
Enterococcus faecium	6.8	0.0	5.5	3.8	10.8	1.1	5.1	4.4
Enterobacter cloacae complex	1.7	6.6	3.4	2.4	4.0	5.1	3.6	4.0
Proteus mirabilis	1.1	3.3	2.0	1.0	0.9	4.7	6.6	3.8
Klebsiella oxytoca	0.7	4.6	1.0	2.0	3.2	2.7	3.1	2.5

Figure S4: Screenshot from GEFAAR, showing the pathogen statistics for the top-10 species stratified into specimens in which they were detected (year: 2022, clinic/unit: all).

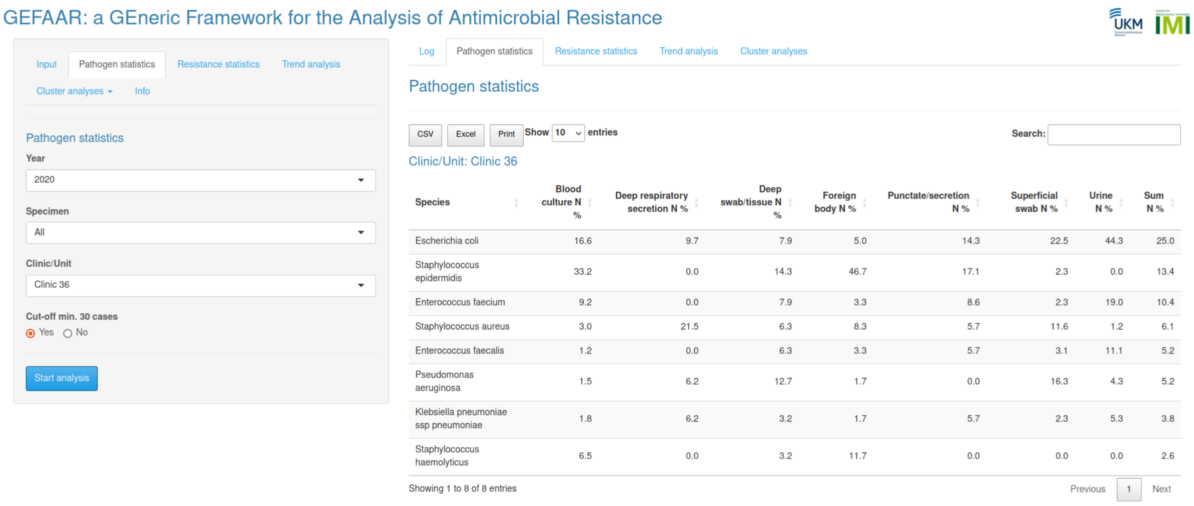

Species	Blood culture N %	Deep respiratory secretion N %	Deep swab/tissue N %	Foreign body N %	Punctate/secretion N %	Superficial swab N %	Urine N %	Sum N %
Escherichia coli	16.6	9.7	7.9	5.0	14.3	22.5	44.3	25.0
Staphylococcus epidermidis	33.2	0.0	14.3	46.7	17.1	2.3	0.0	13.4
Enterococcus faecium	9.2	0.0	7.9	3.3	8.6	2.3	19.0	10.4
Staphylococcus aureus	3.0	21.5	6.3	8.3	5.7	11.6	1.2	6.1
Enterococcus faecalis	1.2	0.0	6.3	3.3	5.7	3.1	11.1	5.2
Pseudomonas aeruginosa	1.5	6.2	12.7	1.7	0.0	16.3	4.3	5.2
Klebsiella pneumoniae ssp pneumoniae	1.8	6.2	3.2	1.7	5.7	2.3	5.3	3.8
Staphylococcus haemolyticus	6.5	0.0	3.2	11.7	0.0	0.0	0.0	2.6

Figure S5: Screenshot from GEFAAR, showing the pathogen statistics for the top-8 species stratified into specimens in which they were detected (year: 2020, clinic/unit: Clinic 36).

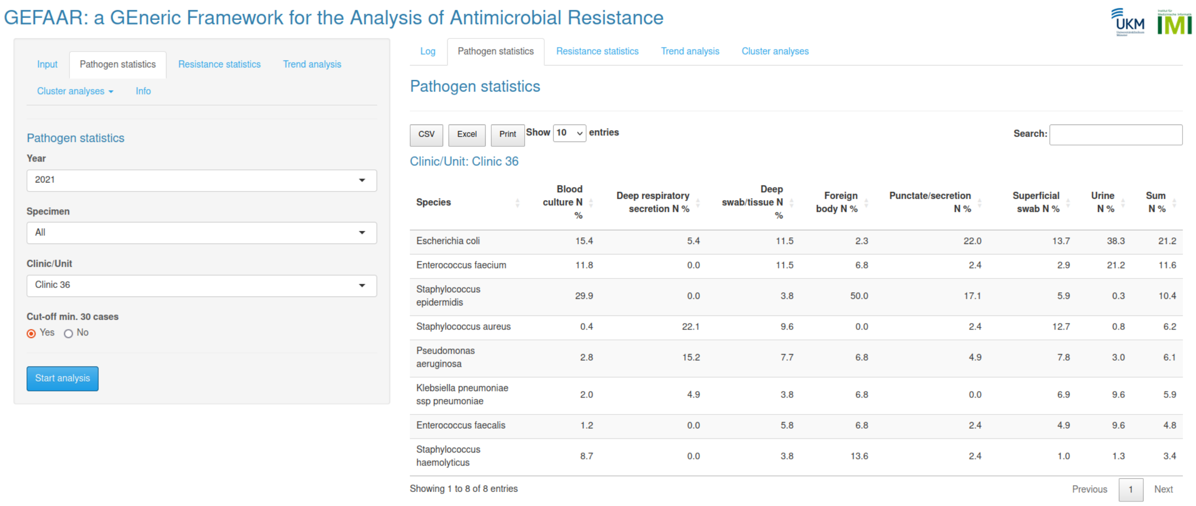

Figure S6: Screenshot from GEFAAR, showing the pathogen statistics for the top-8 species stratified into specimens in which they were detected (year: 2021, clinic/unit: Clinic 36).

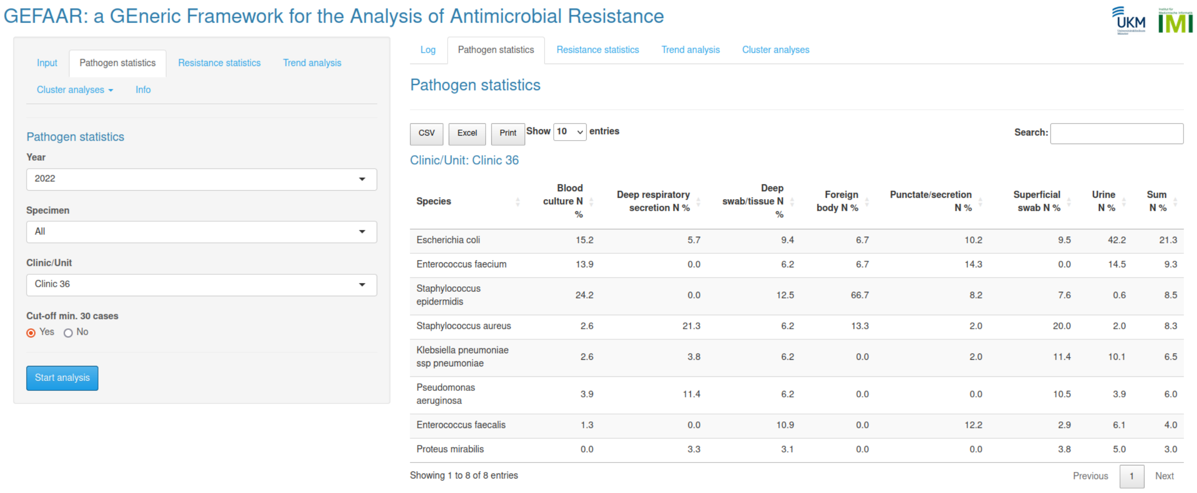

Figure S7: Screenshot from GEFAAR, showing the pathogen statistics for the top-8 species stratified into specimens in which they were detected (year: 2022, clinic/unit: Clinic 36).

2.2 Resistance statistics

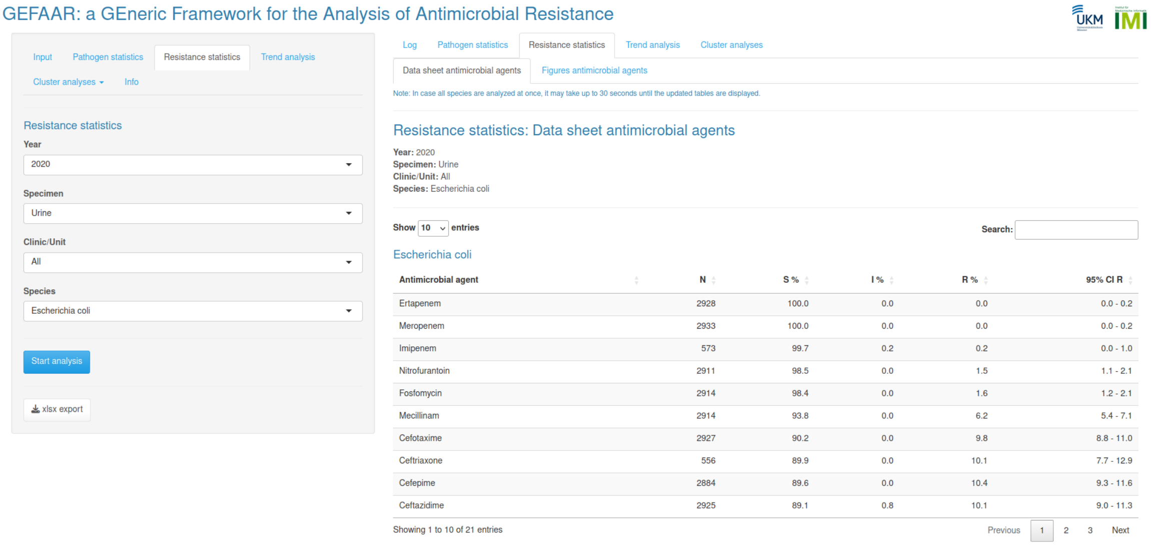

Figure S8: Screenshot from GEFAAR, showing the resistance statistics: data sheet antimicrobial agents for *Escherichia coli* (year: 2020, specimen: urine, clinic/unit: all).

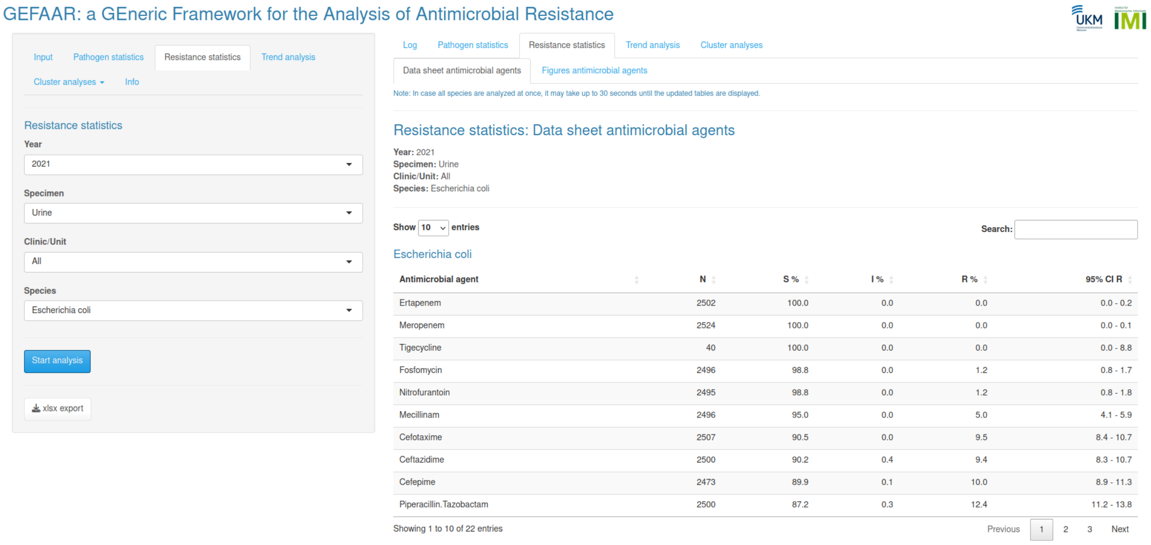

Figure S9: Screenshot from GEFAAR, showing the resistance statistics: data sheet antimicrobial agents for *Escherichia coli* (year: 2021, specimen: urine, clinic/unit: all).

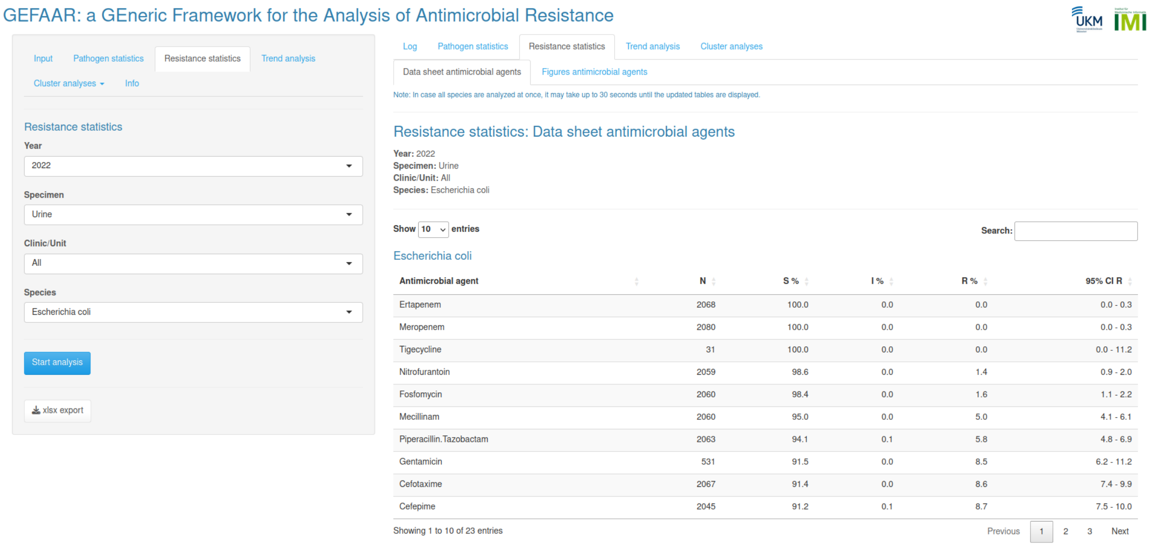

Figure S10: Screenshot from GEFAAR, showing the resistance statistics: data sheet antimicrobial agents for *Escherichia coli* (year: 2022, specimen: urine, clinic/unit: all).

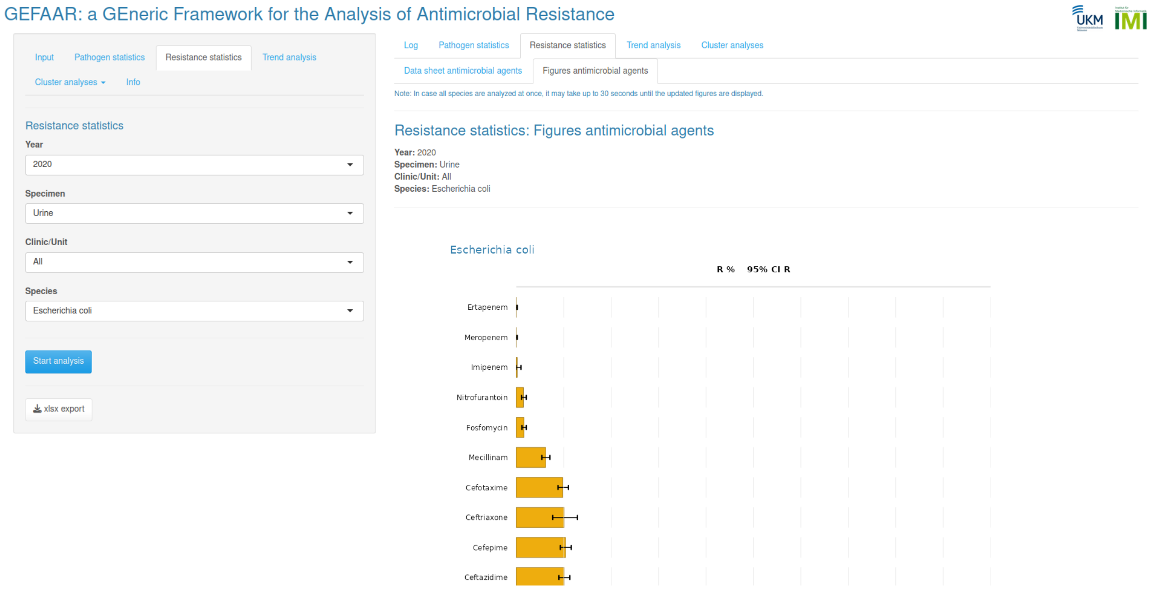

Figure S11: Screenshot from GEFAAR, showing the resistance statistics: figures antimicrobial agents for *Escherichia coli* (year: 2020, specimen: urine, clinic/unit: all).

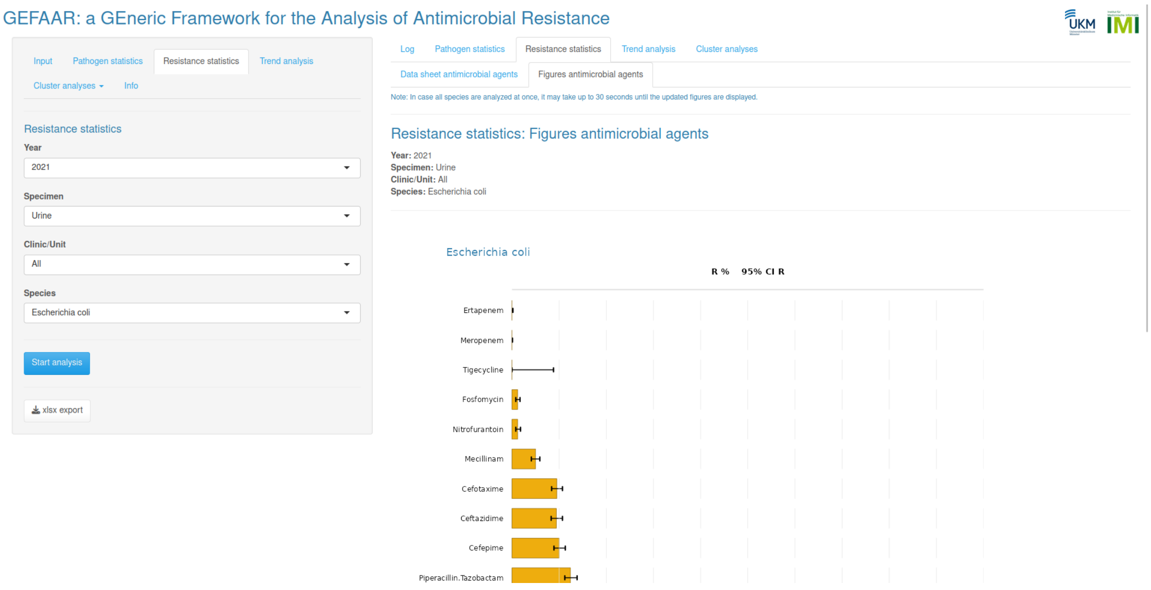

Figure S12: Screenshot from GEFAAR, showing the resistance statistics: figures antimicrobial agents for *Escherichia coli* (year: 2021, specimen: urine, clinic/unit: all).

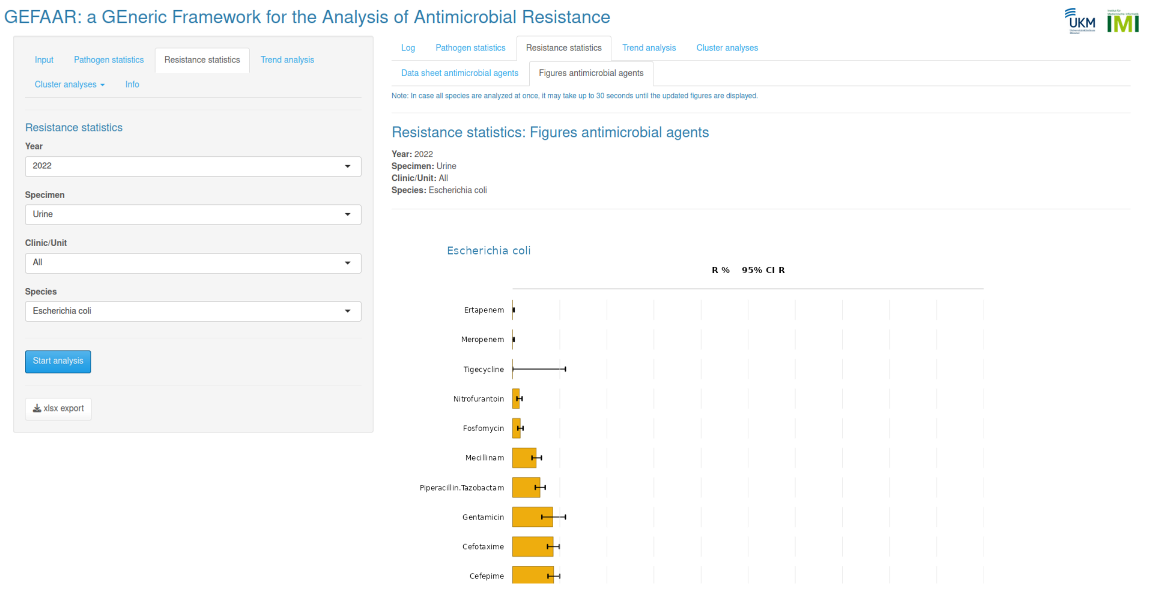

Figure S13: Screenshot from GEFAAR, showing the resistance statistics: figures antimicrobial agents for *Escherichia coli* (year: 2022, specimen: urine, clinic/unit: all).

2.3 Trend analysis

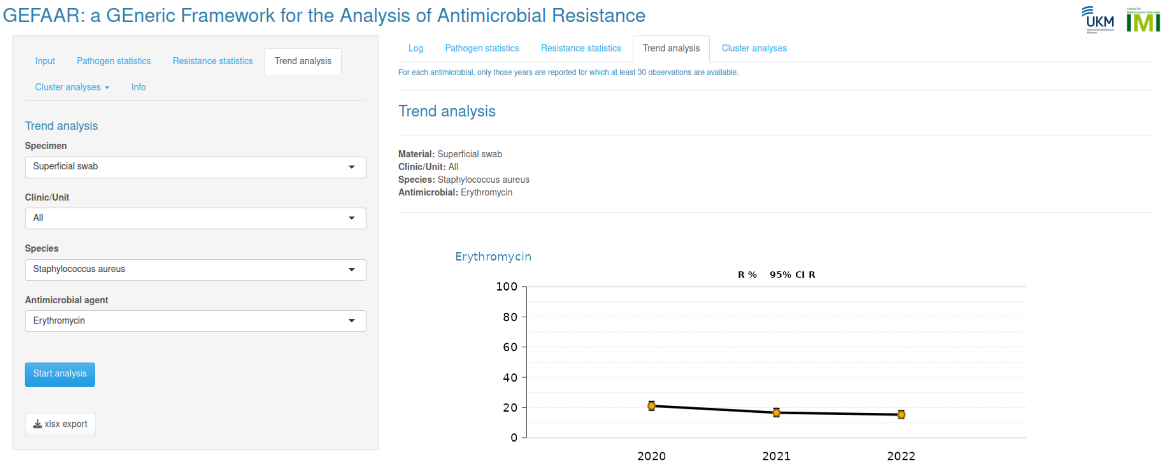

Figure S14: Screenshot from GEFAAR, showing the results of the trend analysis for *Staphylococcus aureus* and erythromycin (specimen: superficial swab, clinic/unit: all).

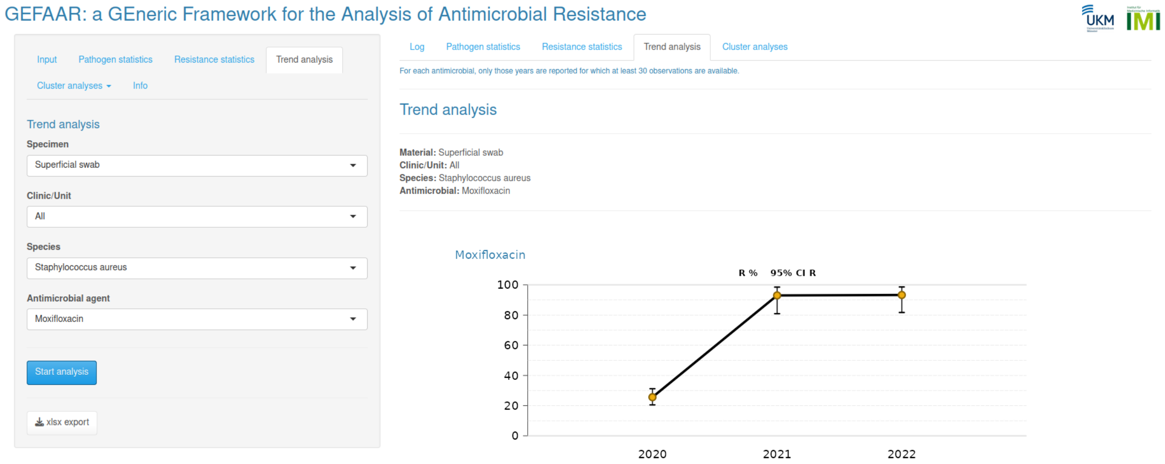

Figure S15: Screenshot from GEFAAR, showing the results of the trend analysis for *Staphylococcus aureus* and moxifloxacin (specimen: superficial swab, clinic/unit: all).

2.4 Cluster analyses

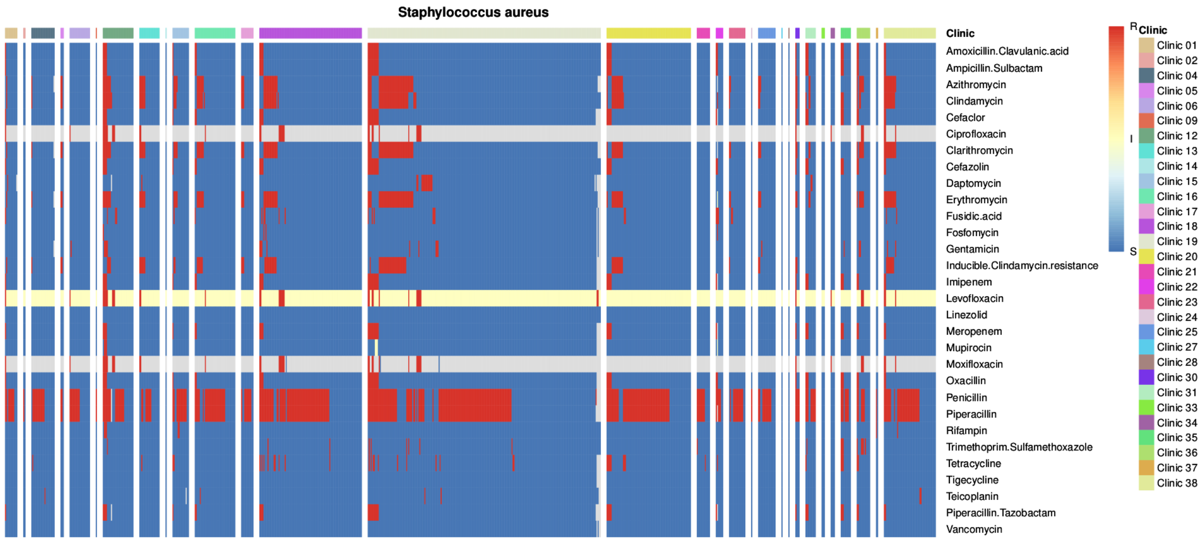

Figure S16: Exported GEFAAR cluster analyses, evaluating data on *Staphylococcus aureus* (year: 2021, specimen: superficial swap). Every column represents one sample, every row one antimicrobial agent. Heatmap ordered by 1) clinic/unit and 2) resistance.

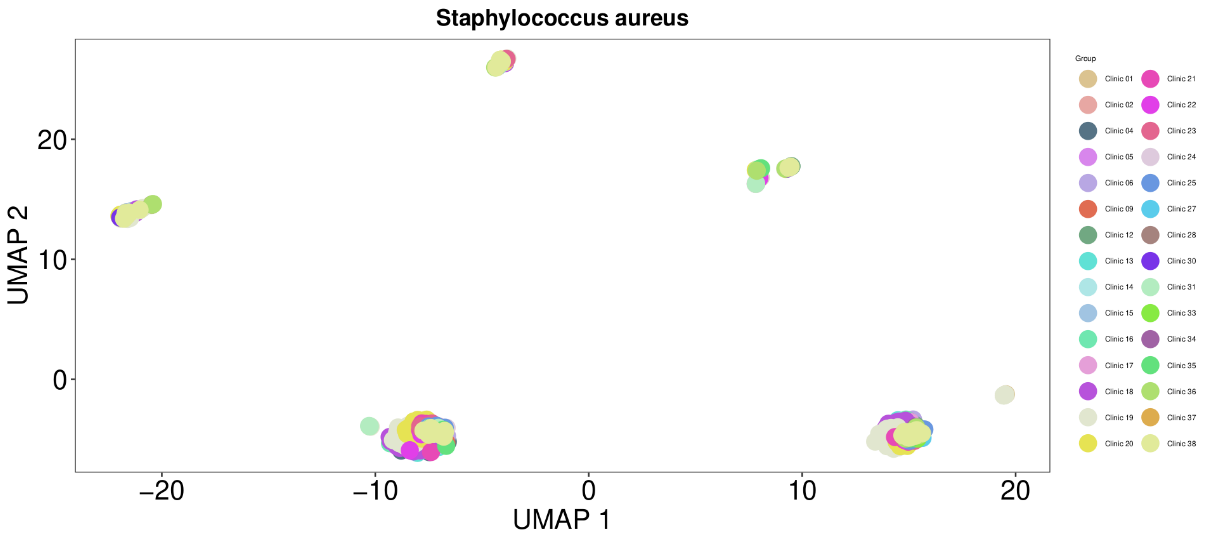

Figure S17: Exported GEFAAR cluster analyses, evaluating data on *Staphylococcus aureus* (year: 2021, specimen: superficial swap). UMAP with colored clinics/units.

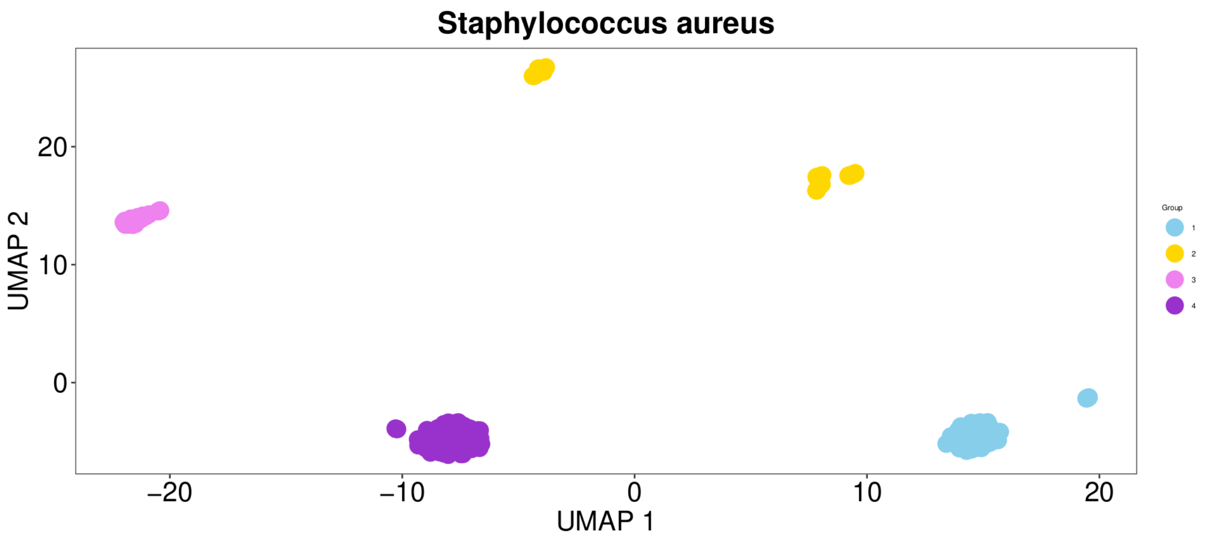

Figure S18: Exported GEFAAR cluster analyses, evaluating data on *Staphylococcus aureus* (year: 2021, specimen: superficial swap). UMAP with colored clusters.

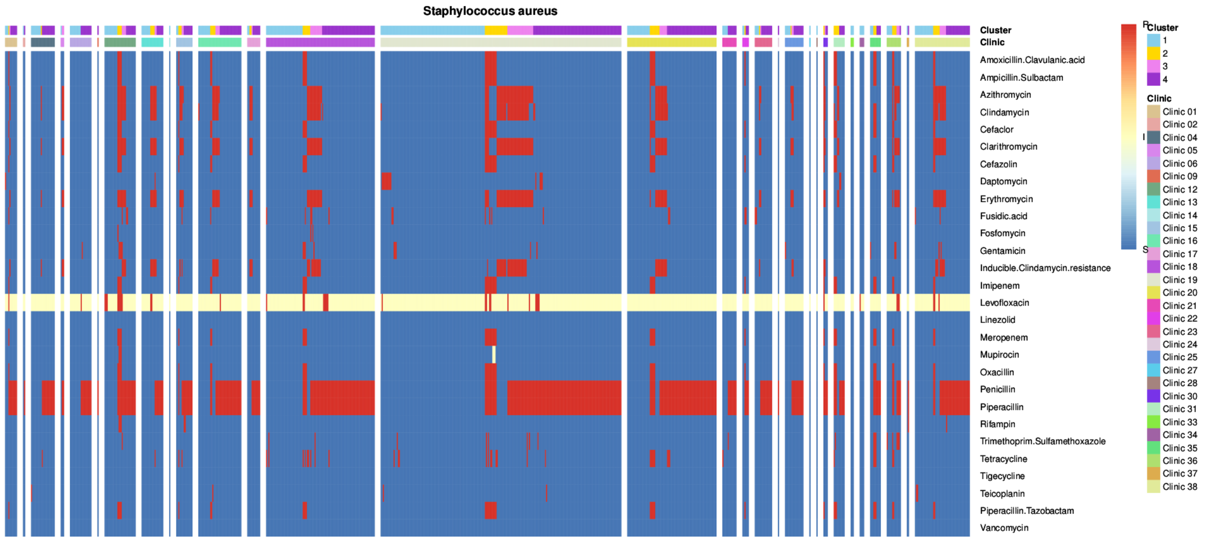

Figure S19: Exported GEFAAR cluster analyses, evaluating data on *Staphylococcus aureus* (year: 2021, specimen: superficial swap). Every column represents one sample, every row one antimicrobial agent. Heatmap showing data ordered by clinics/units (annotated clusters in top row).

3 Legend to supplementary tables

Supplementary Table S1.xlsx: Detailed information on specimens, clinics/units, species and antimicrobial agents in a real dataset analyzed with GEFAAR (samples collected at the University Hospital Münster between 2020 and 2022).
Supplementary Table S2.xlsx: Pathogen statistics generated with GEFAAR, year: 2020, clinic/unit: all.
Supplementary Table S3.xlsx: Pathogen statistics generated with GEFAAR, year: 2021, clinic/unit: all.
Supplementary Table S4.xlsx: Pathogen statistics generated with GEFAAR, year: 2022, clinic/unit: all.
Supplementary Table S5.xlsx: Pathogen statistics generated with GEFAAR, year: 2020, clinic/unit: Clinic 36.
Supplementary Table S6.xlsx: Pathogen statistics generated with GEFAAR, year: 2021, clinic/unit: Clinic 36.
Supplementary Table S7.xlsx: Pathogen statistics generated with GEFAAR, year: 2022, clinic/unit: Clinic 36.
Supplementary Table S8.xlsx: Resistance statistics generated with GEFAAR, year: 2020, specimen: urine, clinic/unit: all, species: *Escherichia coli.*
Supplementary Table S9.xlsx: Resistance statistics generated with GEFAAR, year: 2021, specimen: urine, clinic/unit: all, species: *Escherichia coli.*
Supplementary Table S10.xlsx: Resistance statistics generated with GEFAAR, year: 2022, specimen: urine, clinic/unit: all, species: *Escherichia coli.*
Supplementary Table S11.xlsx: Trend analysis performed with GEFAAR, specimen: superficial swab, clinic/unit: all, species: *Staphylococcus aureus*, antimicrobial agent: erythromycin.
Supplementary Table S12.xlsx: Trend analysis performed with GEFAAR, specimen: superficial swab, clinic/unit: all, species: *Staphylococcus aureus*, antimicrobial agent: moxifloxacin.

4 Legend to supplementary data

Supplementary Data S1.pdf: Cluster analyses per species generated with GEFAAR, year: 2021, specimen: superficial swap, species: *Staphylococcus aureus.* Colors indicate blue: susceptible, yellow: susceptible with induced exposure, red: resistant, grey: no data available.
Supplementary Data S2.pdf: Cluster analyses per clinic/unit generated with GEFAAR, year: 2021, specimen: all, clinic/unit: Clinic 01. Colors indicate blue: susceptible, yellow: susceptible with induced exposure, red: resistant, grey: no data available.
