# Supplementary material for: GEFAAR: a generic framework for the analysis of antimicrobial resistance providing statistics and cluster analyses
Source: Sci Rep. 2023 Oct 7;13:16922. doi: 10.1038/s41598-023-44109-3 (PMC10560266; doi:10.1038/s41598-023-44109-3)
Supplement: Supplementary file 1 — Supplementary Information. [file 41598_2023_44109_MOESM1_ESM.zip › SupplementaryFiles/SupplementaryFiles/Supplementary Data S1.pdf]

Resistance Cluster Analyses

2023−08−22

Universitätsklinikum Münster

Institut für Medizinische Informatik

Independent analysis per species

Heatmap

Data ordered 1) clinic/unit, 2) resistance

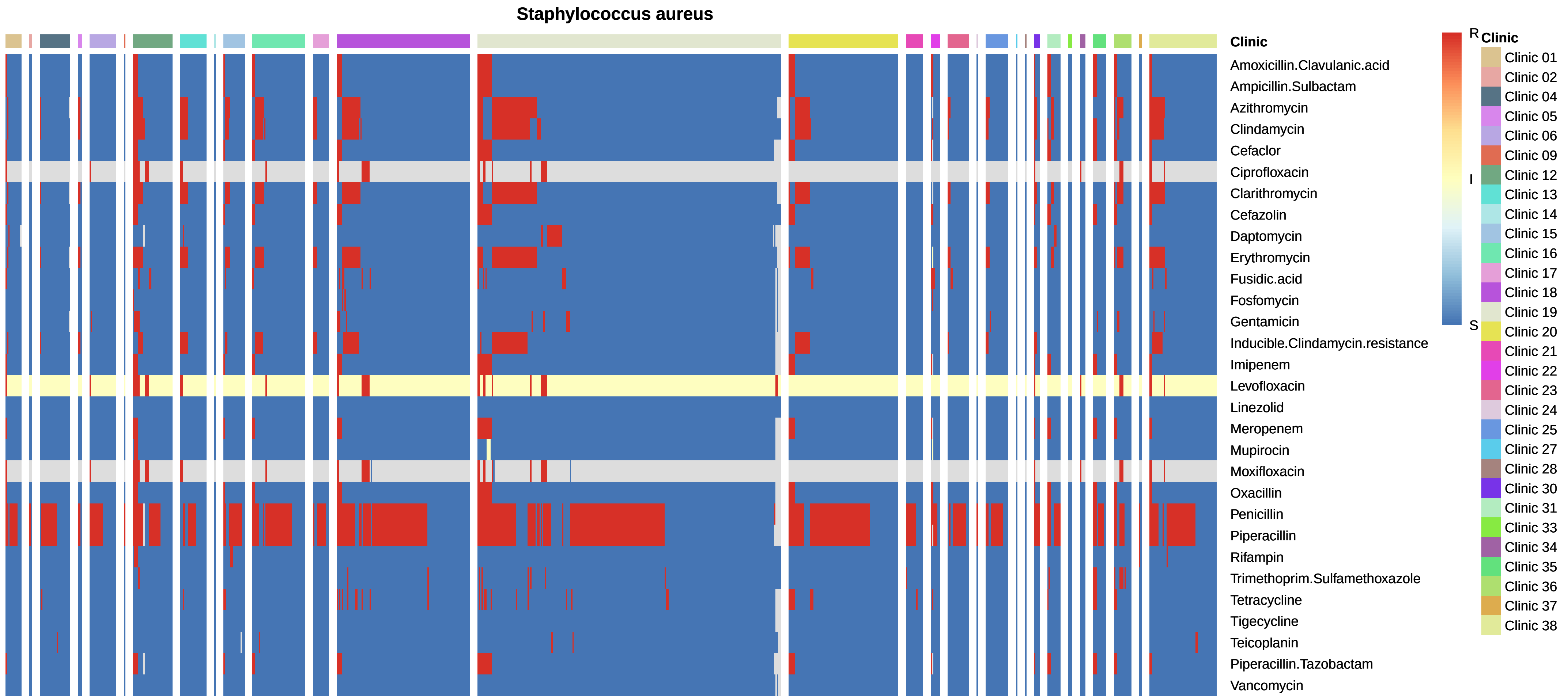
Staphylococcus aureus
Clinic
Amoxicillin.Clavulanic.acid
Ampicillin.Sulbactam
Azithromycin
Clindamycin
Cefaclor
Ciprofloxacin
Clarithromycin
Cefazolin
Daptomycin
Erythromycin
Fusidic.acid
Fosfomycin
Gentamicin
Inducible.Clindamycin.resistance
Imipenem
Levofloxacin
Linezolid
Meropenem
Mupirocin
Moxifloxacin
Oxacillin
Penicillin
Piperacillin
Rifampin
Trimethoprim.Sulfamethoxazole
Tetracycline
Tigecycline
Teicoplanin
Piperacillin.Tazobactam
Vancomycin
R
I
S
Clinic
Clinic 01
Clinic 02
Clinic 04
Clinic 05
Clinic 06
Clinic 09
Clinic 12
Clinic 13
Clinic 14
Clinic 15
Clinic 16
Clinic 17
Clinic 18
Clinic 19
Clinic 20
Clinic 21
Clinic 22
Clinic 23
Clinic 24
Clinic 25
Clinic 27
Clinic 28
Clinic 30
Clinic 31
Clinic 33
Clinic 34
Clinic 35
Clinic 36
Clinic 37
Clinic 38

Heatmap

Data ordered by 1) clinic/unit, 2) date

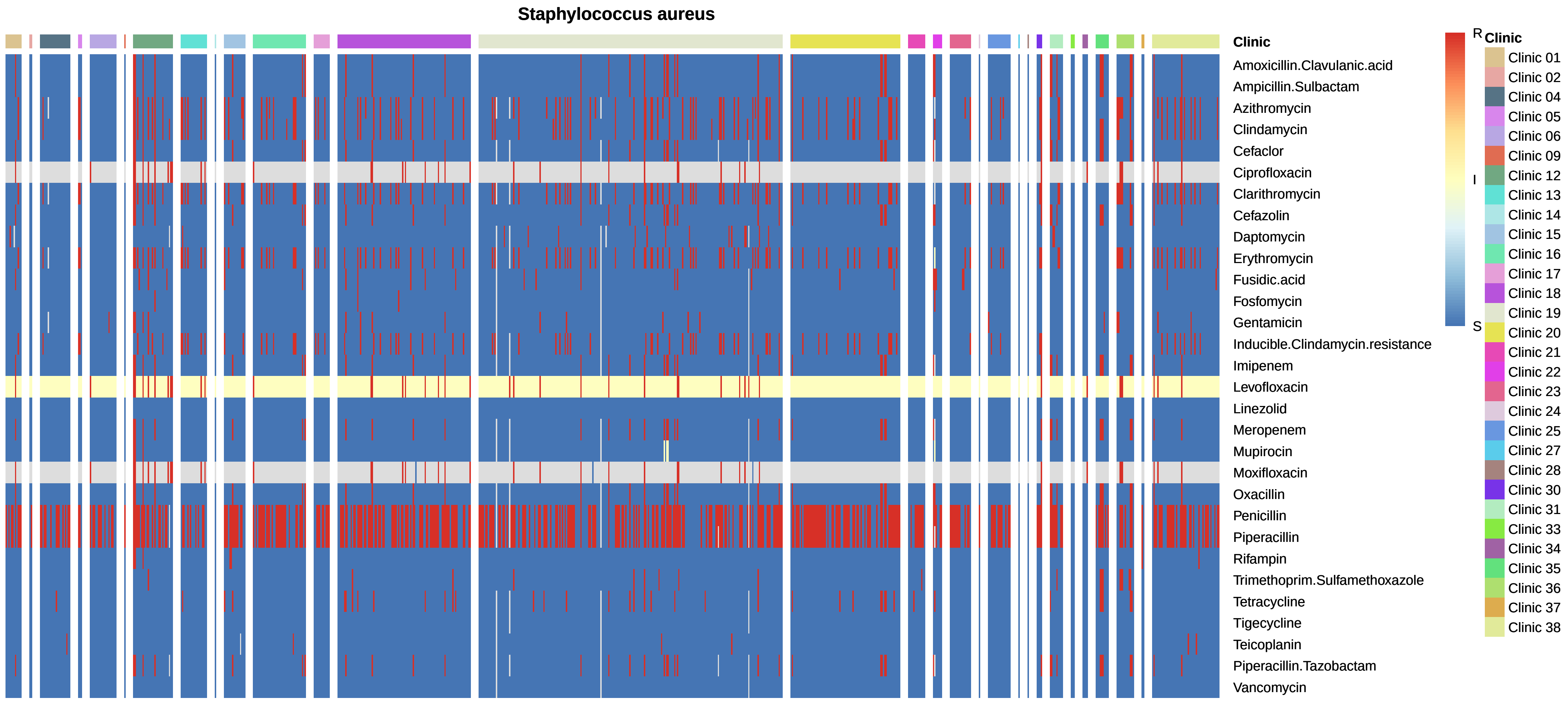
Staphylococcus aureus
Clinic
Amoxicillin.Clavulanic.acid
Ampicillin.Sulbactam
Azithromycin
Clindamycin
Cefaclor
Ciprofloxacin
Clarithromycin
Cefazolin
Daptomycin
Erythromycin
Fusidic.acid
Fosfomycin
Gentamicin
Inducible.Clindamycin.resistance
Imipenem
Levofloxacin
Linezolid
Meropenem
Mupirocin
Moxifloxacin
Oxacillin
Penicillin
Piperacillin
Rifampin
Trimethoprim.Sulfamethoxazole
Tetracycline
Tigecycline
Teicoplanin
Piperacillin.Tazobactam
Vancomycin
R
I
S
Clinic
Clinic 01
Clinic 02
Clinic 04
Clinic 05
Clinic 06
Clinic 09
Clinic 12
Clinic 13
Clinic 14
Clinic 15
Clinic 16
Clinic 17
Clinic 18
Clinic 19
Clinic 20
Clinic 21
Clinic 22
Clinic 23
Clinic 24
Clinic 25
Clinic 27
Clinic 28
Clinic 30
Clinic 31
Clinic 33
Clinic 34
Clinic 35
Clinic 36
Clinic 37
Clinic 38

Heatmap

Hierarchical clustering

Staphylococcus aureus − no clustering possible

UMAP

Plot with colored clinics/units

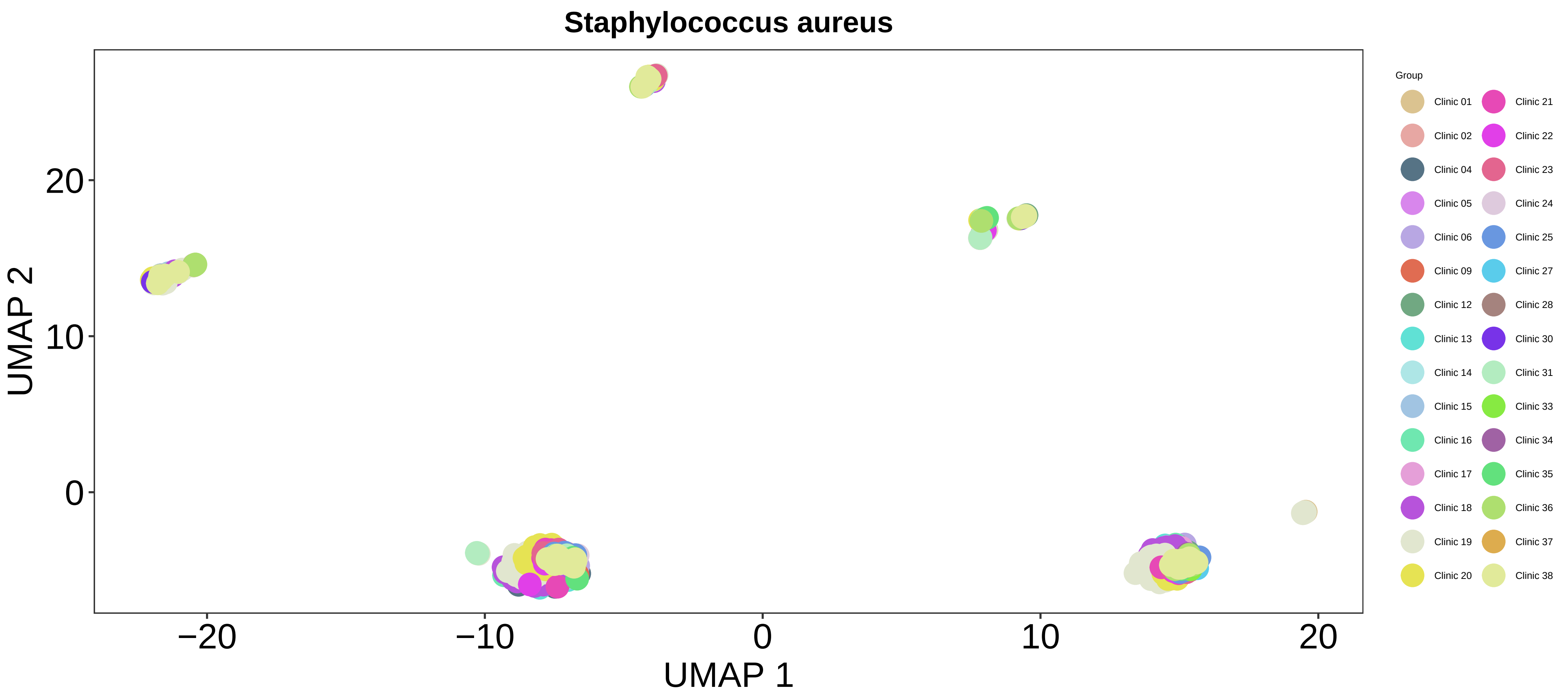
Staphylococcus aureus
UMAP 2
UMAP 1
20
10
0
−20
−10
0
10
20
Group
Clinic 01
Clinic 02
Clinic 04
Clinic 05
Clinic 06
Clinic 09
Clinic 12
Clinic 13
Clinic 14
Clinic 15
Clinic 16
Clinic 17
Clinic 18
Clinic 19
Clinic 20
Clinic 21
Clinic 22
Clinic 23
Clinic 24
Clinic 25
Clinic 27
Clinic 28
Clinic 30
Clinic 31
Clinic 33
Clinic 34
Clinic 35
Clinic 36
Clinic 37
Clinic 38

UMAP

Plot with colored clusters

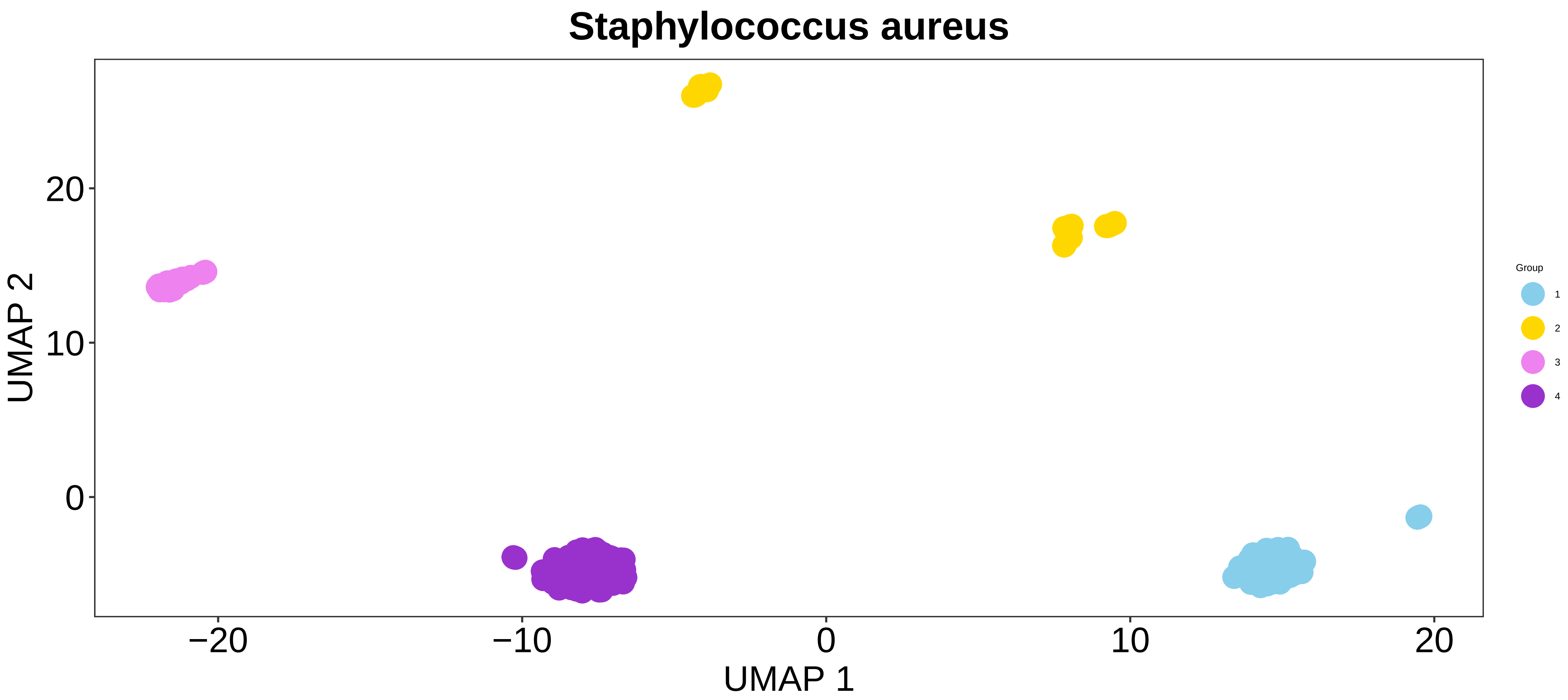
Staphylococcus aureus
UMAP 2
UMAP 1
20
10
0
−20
−10
0
10
20
Group
1
2
3
4

Heatmap

Data ordered by UMAP clusters

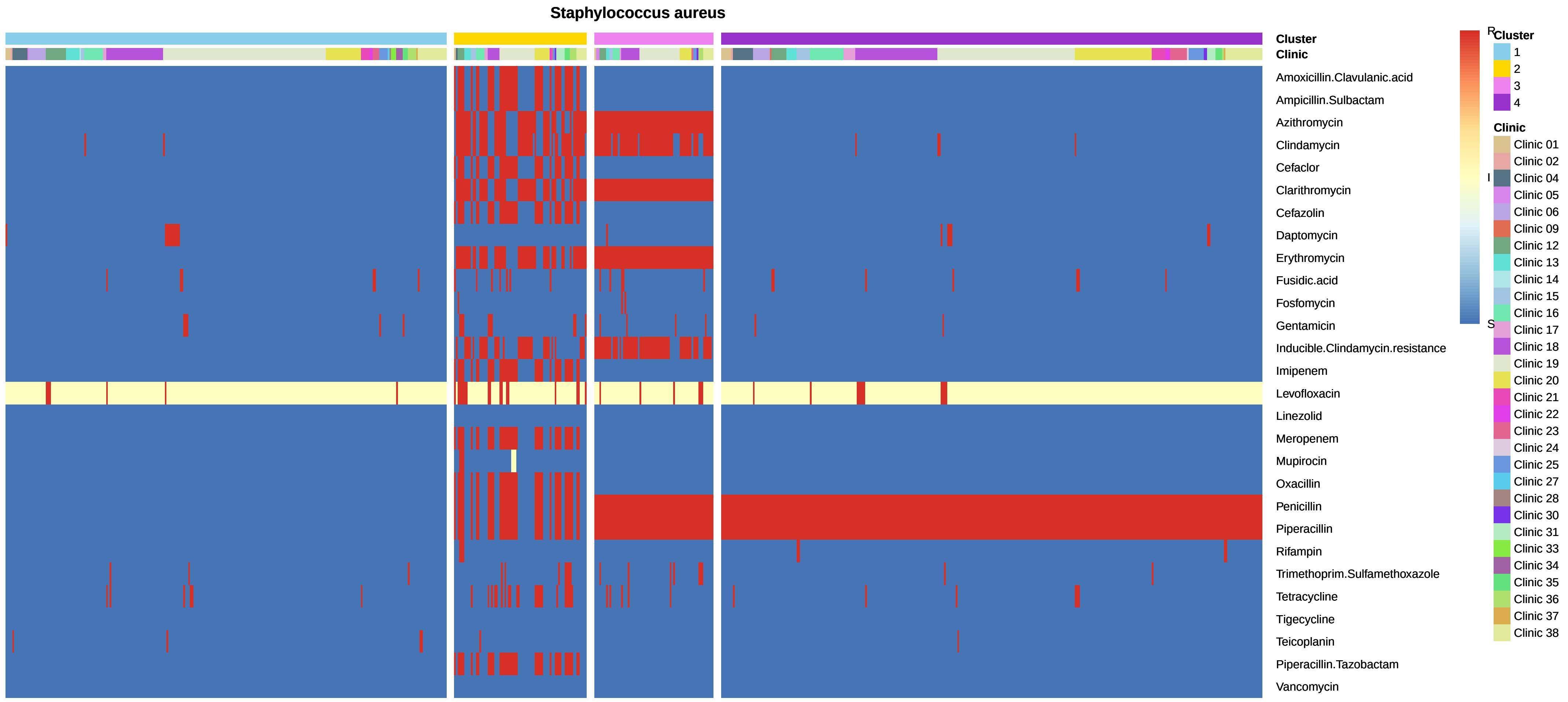
Staphylococcus aureus
Cluster
Clinic
Amoxicillin.Clavulanic.acid
Ampicillin.Sulbactam
Azithromycin
Clindamycin
Cefaclor
Clarithromycin
Cefazolin
Daptomycin
Erythromycin
Fusidic.acid
Fosfomycin
Gentamicin
Inducible.Clindamycin.resistance
Imipenem
Levofloxacin
Linezolid
Meropenem
Mupirocin
Oxacillin
Penicillin
Piperacillin
Rifampin
Trimethoprim.Sulfamethoxazole
Tetracycline
Tigecycline
Teicoplanin
Piperacillin.Tazobactam
Vancomycin
R
I
S
Cluster
1
2
3
4
Clinic
Clinic 01
Clinic 02
Clinic 04
Clinic 05
Clinic 06
Clinic 09
Clinic 12
Clinic 13
Clinic 14
Clinic 15
Clinic 16
Clinic 17
Clinic 18
Clinic 19
Clinic 20
Clinic 21
Clinic 22
Clinic 23
Clinic 24
Clinic 25
Clinic 27
Clinic 28
Clinic 30
Clinic 31
Clinic 33
Clinic 34
Clinic 35
Clinic 36
Clinic 37
Clinic 38

Heatmap

Data ordered by clinic/unit

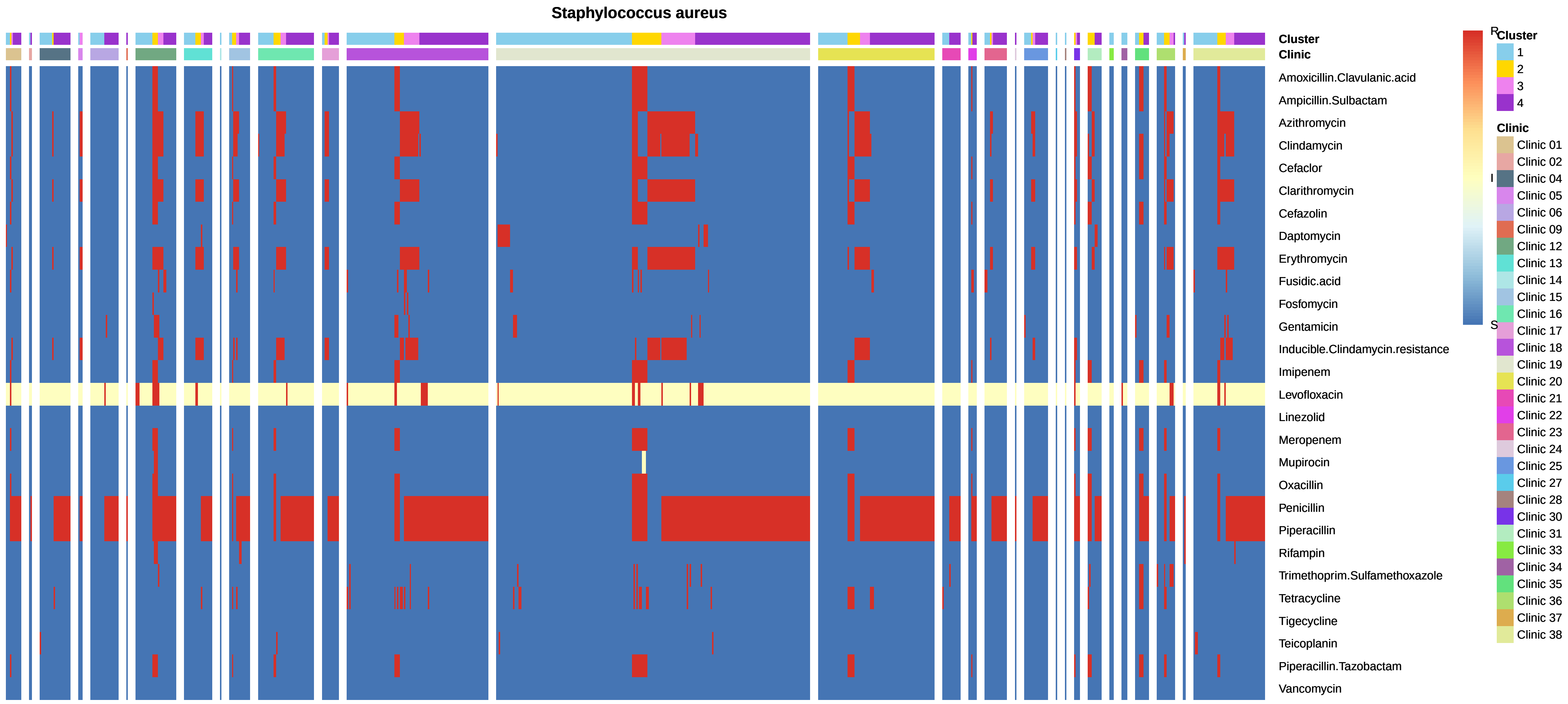

Staphylococcus aureus
Cluster
Clinic
Amoxicillin.Clavulanic.acid
Ampicillin.Sulbactam
Azithromycin
Clindamycin
Cefaclor
Clarithromycin
Cefazolin
Daptomycin
Erythromycin
Fusidic.acid
Fosfomycin
Gentamicin
Inducible.Clindamycin.resistance
Imipenem
Levofloxacin
Linezolid
Meropenem
Mupirocin
Oxacillin
Penicillin
Piperacillin
Rifampin
Trimethoprim.Sulfamethoxazole
Tetracycline
Tigecycline
Teicoplanin
Piperacillin.Tazobactam
Vancomycin
R
I
S
Cluster
1
2
3
4
Clinic
Clinic 01
Clinic 02
Clinic 04
Clinic 05
Clinic 06
Clinic 09
Clinic 12
Clinic 13
Clinic 14
Clinic 15
Clinic 16
Clinic 17
Clinic 18
Clinic 19
Clinic 20
Clinic 21
Clinic 22
Clinic 23
Clinic 24
Clinic 25
Clinic 27
Clinic 28
Clinic 30
Clinic 31
Clinic 33
Clinic 34
Clinic 35
Clinic 36
Clinic 37
Clinic 38
