# Supplementary material for: GEFAAR: a generic framework for the analysis of antimicrobial resistance providing statistics and cluster analyses
Source: Sci Rep. 2023 Oct 7;13:16922. doi: 10.1038/s41598-023-44109-3 (PMC10560266; doi:10.1038/s41598-023-44109-3)
Supplement: Supplementary file 1 — Supplementary Information. [file 41598_2023_44109_MOESM1_ESM.zip › SupplementaryFiles/SupplementaryFiles/Supplementary Data S2.pdf]

Resistance Cluster Analyses

2023−08−22

UKM
Universitätsklinikum
Münster

Institut für
Medizinische Informatik
IMI

Independent analysis per clinic/unit

Heatmap

Data ordered by species

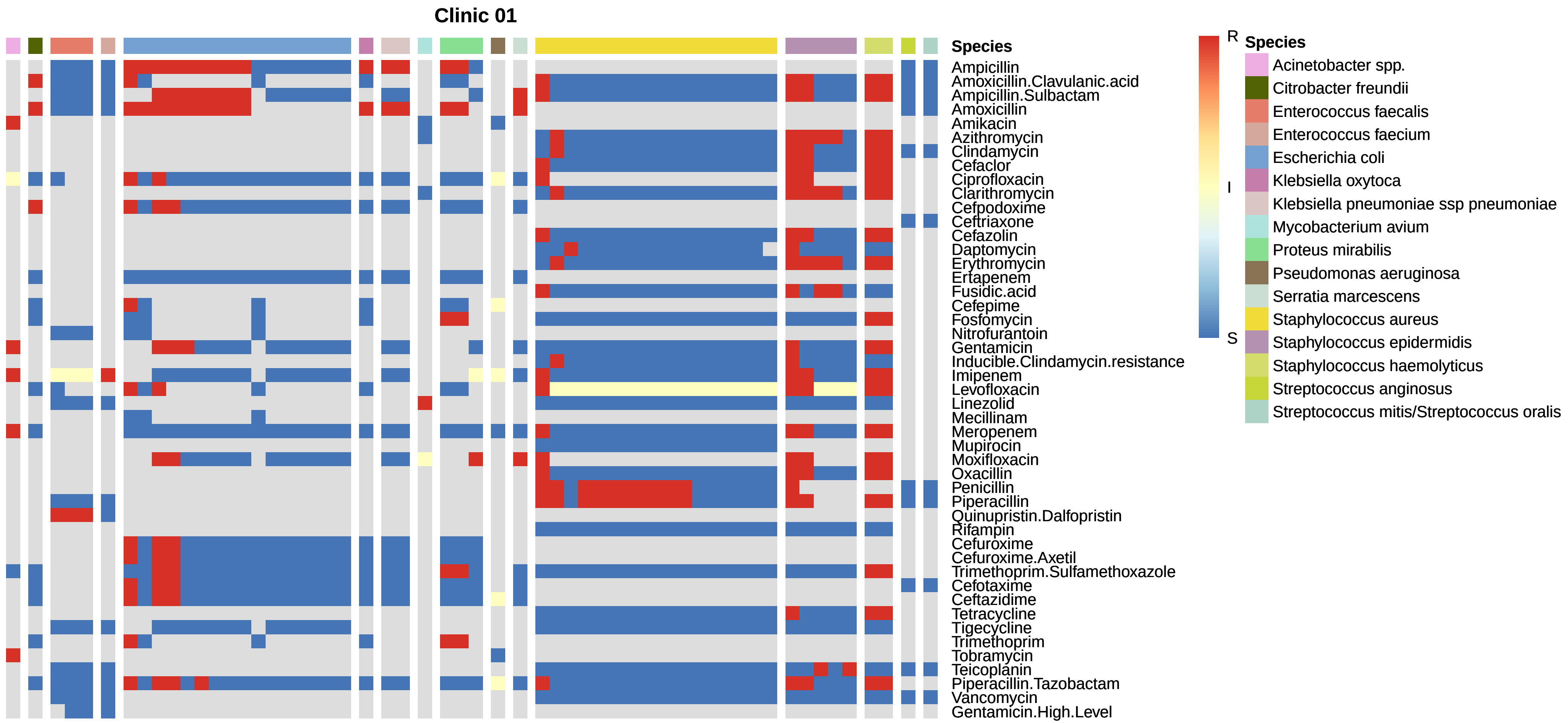
Clinic 01
Species
Ampicillin
Amoxicillin.Clavulanic.acid
Ampicillin.Sulbactam
Amoxicillin
Amikacin
Azithromycin
Clindamycin
Cefaclor
Ciprofloxacin
Clarithromycin
Cefpodoxime
Ceftriaxone
Cefazolin
Daptomycin
Erythromycin
Ertapenem
Fusidic.acid
Cefepime
Fosfomycin
Nitrofurantoin
Gentamicin
Inducible.Clindamycin.resistance
Imipenem
Levofloxacin
Linezolid
Mecillinam
Meropenem
Mupirocin
Moxifloxacin
Oxacillin
Penicillin
Piperacillin
Quinupristin.Dalfopristin
Rifampin
Cefuroxime
Cefuroxime.Axetil
Trimethoprim.Sulfamethoxazole
Cefotaxime
Ceftazidime
Tetracycline
Tigecycline
Trimethoprim
Tobramycin
Teicoplanin
Piperacillin.Tazobactam
Vancomycin
Gentamicin.High.Level
R
I
S
Species
Acinetobacter spp.
Citrobacter freundii
Enterococcus faecalis
Enterococcus faecium
Escherichia coli
Klebsiella oxytoca
Klebsiella pneumoniae ssp pneumoniae
Mycobacterium avium
Proteus mirabilis
Pseudomonas aeruginosa
Serratia marcescens
Staphylococcus aureus
Staphylococcus epidermidis
Staphylococcus haemolyticus
Streptococcus anginosus
Streptococcus mitis/Streptococcus oralis

Heatmap

Hierarchical clustering

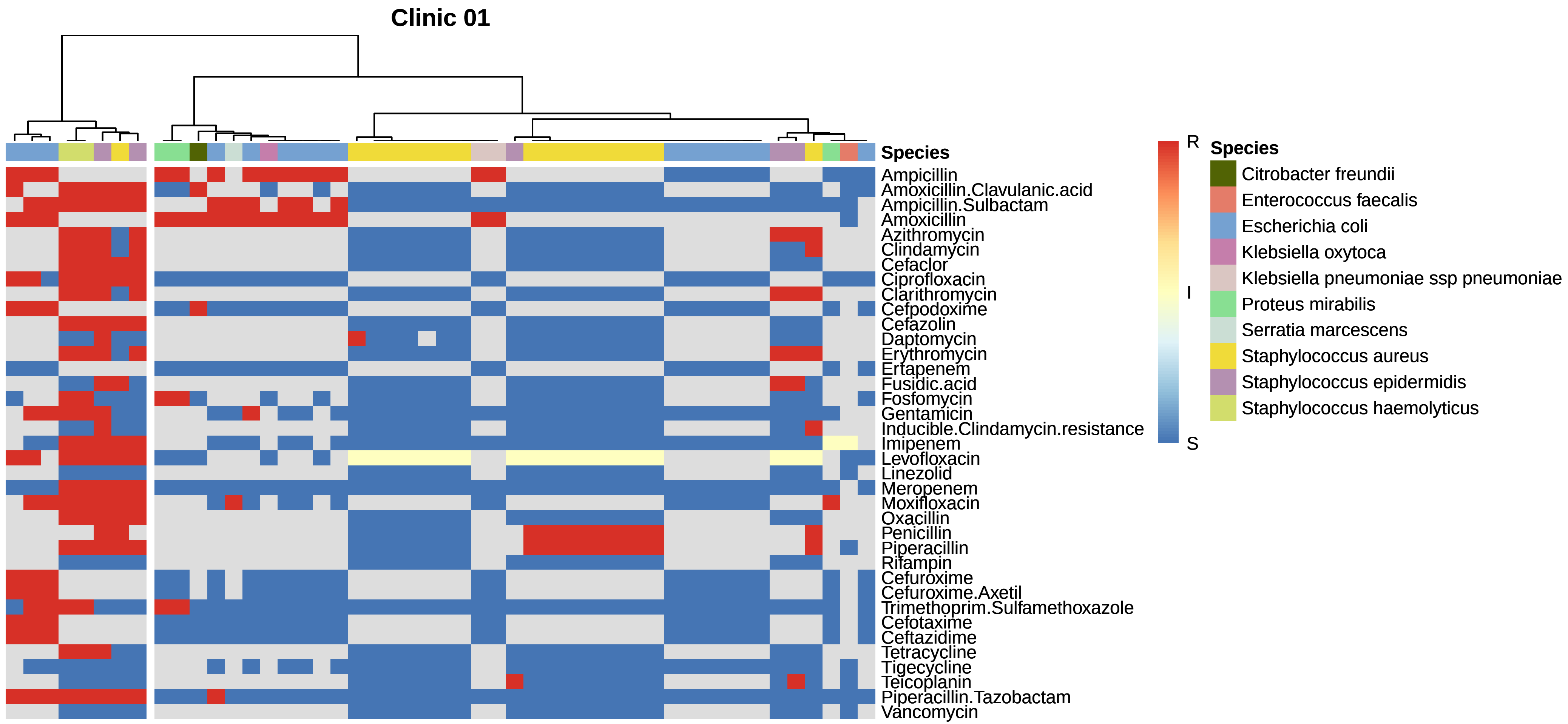
Clinic 01
Species
Ampicillin
Amoxicillin.Clavulanic.acid
Ampicillin.Sulbactam
Amoxicillin
Azithromycin
Clindamycin
Cefaclor
Ciprofloxacin
Clarithromycin
Cefpodoxime
Cefazolin
Daptomycin
Erythromycin
Ertapenem
Fusidic.acid
Fosfomycin
Gentamicin
Inducible.Clindamycin.resistance
Imipenem
Levofloxacin
Linezolid
Meropenem
Moxifloxacin
Oxacillin
Penicillin
Piperacillin
Rifampin
Cefuroxime
Cefuroxime.Axetil
Trimethoprim.Sulfamethoxazole
Cefotaxime
Ceftazidime
Tetracycline
Tigecycline
Teicoplanin
Piperacillin.Tazobactam
Vancomycin
R
I
S
Species
Citrobacter freundii
Enterococcus faecalis
Escherichia coli
Klebsiella oxytoca
Klebsiella pneumoniae ssp pneumoniae
Proteus mirabilis
Serratia marcescens
Staphylococcus aureus
Staphylococcus epidermidis
Staphylococcus haemolyticus
